# Screening and Identification of Four Prognostic Genes Related to Immune Infiltration and G-Protein Coupled Receptors Pathway in Lung Adenocarcinoma

**DOI:** 10.3389/fonc.2020.622251

**Published:** 2021-02-08

**Authors:** Yan Wang, Liwei Qiu, Yu Chen, Xia Zhang, Peng Yang, Feng Xu

**Affiliations:** ^1^ Department of Emergency Medicine, The First Affiliated Hospital of Soochow University, Suzhou, China; ^2^ Department of Emergency Medicine, Affiliated Hospital of Nantong University, Nantong, China; ^3^ Research Center of Clinical Medicine, Affiliated Hospital of Nantong University, Nantong, China

**Keywords:** lung adenocarcinoma, tumor immune infiltration, single sample gene set enrichment analysis, weighted correlation network analysis, prognosis

## Abstract

**Background:**

Lung adenocarcinoma (LUAD) is a common malignant tumor with the highest morbidity and mortality worldwide. The degree of tumor immune infiltration and clinical prognosis depend on immune-related genes, but their interaction with the tumor immune microenvironment, the specific mechanism driving immune infiltration and their prognostic value are still not very clear. Therefore, the aim of this work was focused on the elucidation of these unclear aspects.

**Methods:**

TCGA LUAD samples were divided into three immune infiltration subtypes according to the single sample gene set enrichment analysis (ssGSEA), in which the associated gene modules and hub genes were screened by weighted correlation network analysis (WGCNA). Four key genes related to immune infiltration were found and screened by differential expression analysis, univariate prognostic analysis, and Lasso-COX regression, and their PPI network was constructed. Finally, a Nomogram model based on the four genes and tumor stages was constructed and confirmed in two GEO data sets.

**Results:**

Among the three subtypes—high, medium, and low immune infiltration subtype—the survival rate of the patients in the high one was higher than the rate in the other two subtypes. The four key genes related to LUAD immune infiltration subtypes were CD69, KLRB1, PLCB2, and P2RY13. The PPI network revealed that the downstream genes of the G-protein coupled receptors (GPCRs) pathway were activated by these four genes through the S1PR1. The risk score signature based on these four genes could distinguish high and low-risk LUAD patients with different prognosis. The Nomogram constructed by risk score and clinical tumor stage showed a good ability to predict the survival rate of LUAD patients. The universality and robustness of the Nomogram was confirmed by two GEO datasets.

**Conclusions:**

The prognosis of LUAD patients could be predicted by the constructed risk score signature based on the four genes, making this score a potential independent biomarker. The screening, identification, and analysis of these four genes could contribute to the understanding of GPCRs and LUAD immune infiltration, thus guiding the formulation of more effective immunotherapeutic strategies.

## Introduction

Lung cancer is a common malignant tumor worldwide. More than 2 million new lung cancer cases and nearly 1.8 million deaths occurred in 2018 (1). The incidence and mortality of lung cancer rank first among all malignant tumors (2). Lung cancer is mainly divided into two pathological forms: small cell lung cancer (SCLC) and non-small cell lung cancer (NSCLC), the latter being the prevalent one, accounting for 85~90% of lung cancers. NSCLC is mainly divided into lung squamous cell carcinoma and lung adenocarcinoma (LUAD), which is the most common lung cancer subtype. About half of patients with LUAD are at an advanced stage at the time of diagnosis, with an average 5-year survival rate of only 4% (3).

In recent years, immunotherapy based on blocking strategies of immune checkpoints (PD-1/PD-L1/CTLA-4) revealed considerable survival benefits in a variety of solid tumors, including LUAD (4–6), although only a small number of tumor patients showed a sustained response to immunotherapy (7). More and more evidence is available on the importance of tumor microenvironment (TME) in tumor proliferation, metastasis, and resistance to immunotherapy (8–10). The interaction between tumor cells and immune modulatory factors in TME is the key factor influencing the positive response of tumor patients to immunotherapy (11, 12).

TME represents the environment around tumor cells, composed of extracellular matrix, blood vessels and immune cells, all playing an important role in tumor immunity and closely related to tumor progression and treatment results (13, 14). Many studies confirmed the involvement of TME in the response of immunotherapy and resistance to different drugs in different types of cancer, including LUAD, thus compromising the prognosis of patients (15–17).

However, at present, most of the studies on prognostic models of LUAD only have focused on the changes of gene expression, while the relationship between differentially expressed genes and different levels of immune infiltration is not yet well understood because of the limitation of the existing literature. In addition, the articles available did not study the specific mechanism involved in the aforementioned relationship, and there are too many screening genes involved in the models that maybe lead to overfitting, thus preventing satisfactory results from being achieved (18–20). In this study, our aim was to identify genes that are highly associated with different immune infiltration conditions in LUAD using the RNA-seq data downloaded from The Cancer Genome Atlas (TCGA) database, and to analyze the potential pathways in which these genes are involved when regulating the immune infiltration by the use of bioinformatics. Furthermore, a prognostic model was constructed based on these genes, the applicability, and the value of the model in LUAD were evaluated, and the universality of the model was investigated by the external validation of multiple data sets from the Gene Expression Omnibus (GEO) database. The results of this study might highlight a potentially useful systematic and comprehensive screening process of immune infiltration-related genes resulting in the discovery of potential targets for immunotherapy, and a model for predicting the prognosis of LUAD.

## Material and Methods

### LUAD Data Download

The RNA-seq data and clinical information of 487 LUAD patients were downloaded from the TCGA database through the GDC website (https://portal.gdc.cancer.gov/). The RNA-seq data include HTSeq-FPKM data and counts data (the latter used only for the identification of differentially expressed genes). After data cleaning consisting of the removal of the repeated samples, paraffin section samples and samples with missing prognostic data, a total of 426 LUAD samples with complete clinical data were included in this study (Training cohort, **Table 1**). Five microarray datasets were downloaded from the GEO database to confirm the results, and two of them were used as validation cohorts by incorporating them into the external validation of the prognostic model (**Tables 1** and **2**).

**Table 1:** Clinical data of LUAD patients.

	Training Cohort (TCGA)	Validation Cohort 1 (GSE 41271)	Validation Cohort 2 (GSE 72094)
Number of LUAD	426	182	398
Age (years old)			
Medium	66	64	69
Average	65	64	69
Min	33	30	38
Max	88	86	89
Gender			
Male	190	92	176
Female	236	90	222
Tumor Stage			
Stage I	224	101	254
Stage II	107	28	67
Stage III and IV	95	53	77
T Stage		not provided	not provided
T1 and T2	368		
T3 and T4	55		
TX	3		
M Stage		not provided	not provided
M0	283		
M1	22		
MX	121		
N Stage		not provided	not provided
N0	270		
N1	82		
N2 & N3	63		
NX	11		

**Table 2:** Validation cohorts from GEO database.

Data Set	Pathology	Type	Platform	Samples	LUAD
GSE 41271	NSCLC	Microarray	GPL6884	275	182
GSE 72094	LUAD	Microarray	GPL15048	442	398
GSE 50081	NSCLC	Microarray	GPL570	181	127
GSE 68465	LUAD	Microarray	GPL96	462	442
GSE 42127	NSCLC	Microarray	GPL6884	176	133

### Identification of the Immune Infiltration Subtypes in LUAD

A total of 29 gene sets associated to tumor immune cells and immune functions were obtained from several articles (21–25). The “GSVA” R package was used to perform the single sample gene set enrichment analysis (ssGSEA) on these 29 immune-related gene data sets to obtain the immune infiltration score in each sample to allow the clustering of all samples into three immune infiltration subtypes (26). Next, the “ESTIMATE” R package was used to calculate the tumor purity (representing the proportion of cancer cells in the tumor), the immune score (representing the infiltration of immune cells in the tumor), the stromal score (capturing the presence of stroma in the tumor), and the ESTIMAT score (sum of immune and stromal score) of each sample, which were included in the heat map of ssGSEA to verify the relationship between these subtypes and tumor purity (27). Finally, the relative fractions of 22 kinds of tumor immune cells were calculated in the subgroups by a deconvolution algorithm using the “CIBERSORT” R package (28).

### Screen of Coordinated Expression Genes Related With Immune Infiltration

The coordinated expression genes related with clinical traits and immune infiltration subtypes of LUAD were screened out using weighted correlation network analysis (WGCNA) of the “WGCNA” R package (29). A scale-free topological network model was built using 18,748 genes obtained after data filtering (removing the duplicate genes and genes with average FPKM <5 in total samples), by calculating the correlation of the expression of these genes among each other. TOM-based differences through dynamic tree cutting were used to form modules related to traits (patients’ clinical phenotype and immune infiltration). The minimum module size in this WGCNA network was set at 30 and the height was set at 0.25. The coordinated expression gene network was plotted based on the evaluation of the module eigengenes (MEs), gene significance (GS), and module membership (MM).

### Construction of the Protein-Protein Interaction (PPI) Network and GO/KEGG Enrichment Analysis

Top 300 gene pairs in the two modules screened by WGCNA with the highest GS in each module were selected to construct the PPI network using the “Cytoscape” software (Version 3.8.0). The immune infiltration key genes finally screened were analyzed by inputting the names into the “STRING” website (https://STRING-db.org/) (30). The minimum required interaction score was set as 0.4 during the STRING PPI analysis, while the max number of interactions was set as no more than 10, the line color was set to indicate the type of interaction and the node color was set to indicate the gene ontology (GO) terms to which the gene belongs. The “ClusterProfiler” R package was used to perform GO and the Kyoto Encyclopedia of Genes and Genomes (KEGG) enrichment analysis (31). Three aspects were mainly investigated by the GO analysis: biological process, molecular function, and cellular component.

### Construction and Validation of the Risk Score Signature and Nomogram Model

The “Least Absolute Shrinkage and Selection Operator” (Lasso)-Cox algorithm in “glmnet” R package was used to screen the independent prognostic genes (32) and the selected ones were subsequently used to construct a risk score signature according to a coefficient calculated by Lasso regression. Univariate/multivariate COX regression analysis was performed, a Nomogram prediction model was established, and an external validation in GEO datasets was carried out, all of them using the “Survival” R package. The Time-dependent receiver operating characteristic (ROC) curve was plotted by the “survivalROC” R package.

### Statistical Analysis

The differentially expressed genes (based on counts data) between high immune infiltration subtype and medium-low immune infiltration subtype were found using the “DEseq2” R package. Genes with a false discovery rate (FDR) less than 0.05 were defined as differentially expressed genes (DEGs). The “networkD3” R package was used to perform the principal component analysis (PCA) and to plot the Sankey diagram. The Kaplan-Meier survival curves were tested by the log-rank method, the data in two groups were compared by Mann–Whitney test, the data in multiple groups were compared by Wilcox test, and the Pearson correlation coefficient test was performed to the linear relationship between two quantitative measures. The *P* values (or adjusted *P*-values, *P*adj) of all statistical analysis were calculated using R software (Vision 4.0.2), and a value less than 0.05 was considered statistically significant.

## Results

### Immune Infiltration Subtypes in LUAD

**Figure 1:**
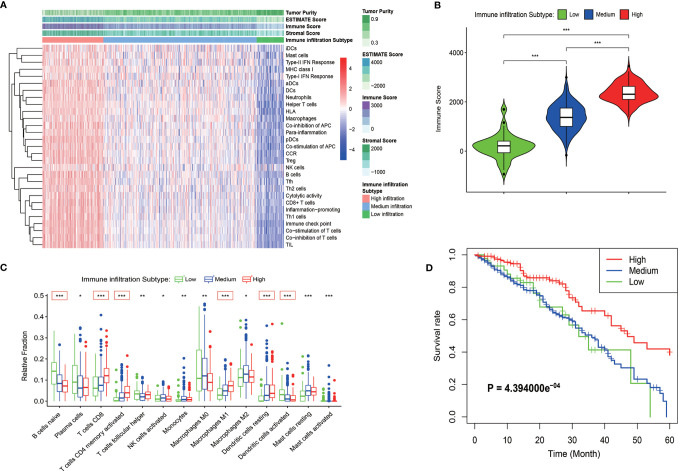
Identification of the immune infiltration subtypes in LUAD in the TCGA cohort. **(A)** According to 29 immune-related gene sets, three immune infiltration subtypes were identified in LUAD samples by ssGSEA. The results of the ESTIMATE algorithm are also shown in the heatmap. **(B)** The immune score calculated by the ESTIMATE algorithm in different immune infiltration subtypes. **(C)** Relative fractions of 14 immune cells in different immune infiltration subtypes. The red box indicated that the relative expression of immune cells increased or decreased in the three immune infiltration subtypes. **(D)** Kaplan-Meyer survival curve of the three immune infiltration subtypes in the TCGA cohort. *P < 0.05; **P < 0.01; ***P < 0.001.

Immune cells and immune-related pathways cause a different immune infiltration and anti-tumor effect in the immune TME. The immune infiltration status of the transcriptome LUAD data was evaluated using the ssGSEA algorithm. A total of 575 immune-related genes included in 29 immune-related gene sets were used to evaluate the immune infiltration in LUAD. The samples in the training cohort were clustered into three immune infiltration subtypes using the hierarchical and K-means clustering method as follows: 44 cases with low immune infiltration, 265 cases with medium immune infiltration, and 118 cases with high immune infiltration (**Figures 1A** and **S1A**). The relationship between the immune infiltration status and tumor purity was evaluated using the ESTIMATE algorithm, and the tumor purity, ESTIMATE score, stromal score, and immune score of the TCGA cohort were calculated. The heatmap and violin plots revealed that the LUAD cases with high immune infiltration were often with lower tumor purity and higher ESTIMATE, stromal and immune scores compared with the cases with low and medium immune infiltration (**Figures 1A, B** and **S1B–D**). The CIBERSORT algorithm was used to quantify the relative fractions of immune cells in the LUAD samples to evaluate more accurately the infiltration status of immune cells in the different immune infiltration subtypes (**Figure S1E**). Fourteen types among the 22 types of immune cells defined by the algorithm were present in different counts in the immune infiltration subtypes (*P* < 0.05). Six types of immune cells, such as immature B cells, CD8 T cells, activated memory CD4 T cells, M1 macrophage cells, and dendritic cells, have the highest significances of infiltration degree in different immune infiltration subtypes (*P* < 0.001), and showed a progressive increasing or decreasing trend (**Figure 1C**). The Kaplan-Meyer survival curve of the three immune infiltration subtypes in the TCGA cohort showed that the 5-year overall survival rate of the high immune infiltration subtype was significantly higher than that in the medium and low immune infiltration subtypes (*P* = 4.394000e-4, **Figure 1D**).

### Screen of the Coordinated Expression Genes Related With Immune Infiltration by WGCNA

**Figure 2:**
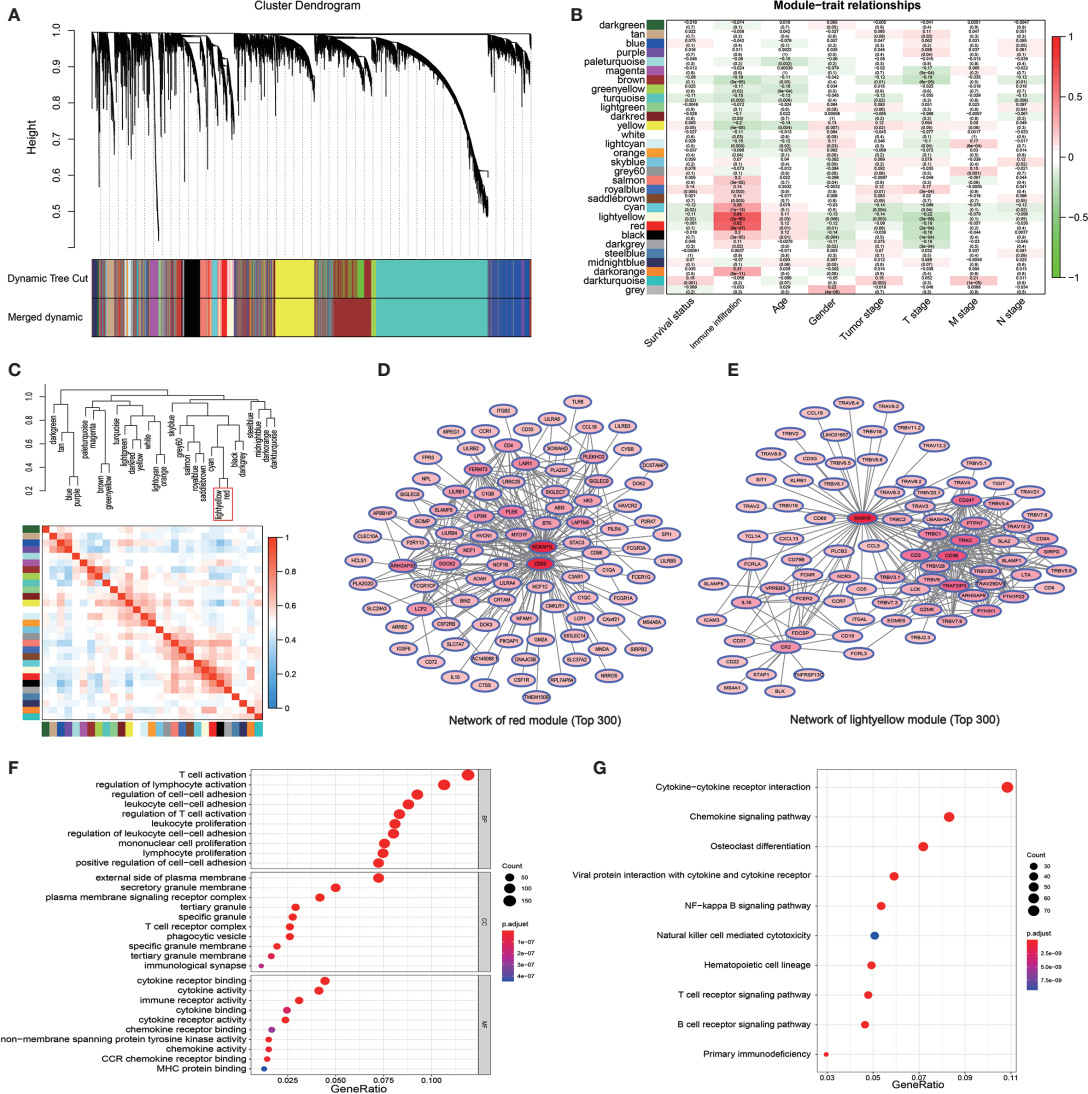
Detection of the immune infiltration related modules and genes by WGCNA. **(A)** The gene dendrogram obtained by the different clusters based on scale-free topological network corresponding to the module represented by the colors in the row. Each colored module contains a group of highly coordinated expression genes. **(B)** Relationship between the gene modules and the different clinical traits of LUAD in the TCGA cohort. **(C)** Dendrogram and heatmap of the correlation of modules. The red box shows the high correlation between the red module and light-yellow module. **(D, E)** The PPI network of the top 300 gene pairs with the highest GS in the red module **(D)** and in the light-yellow module **(E)**. The intensity of the red color of the nodes represents the number of coordinated expression genes in this node gene. **(F)** GO enriched analysis of the coordinated expression genes in red and light-yellow modules. **(G)** KEGG pathway analysis of the coordinated expression genes in red and light-yellow modules.

The WGCNA algorithm was used to construct a weighted correlation gene network to screen the coordinated expression genes associated with immune infiltration. A total of 18,748 genes were included in the WGCNA analysis after the removal of the duplicate genes and genes with low expression. WGCNA clustered all these genes into 14 gene modules by selecting the appropriate soft threshold (**Figures S2A, B**) according to the coordinated pattern (**Figure 2A**). These gene modules were associated with both the clinical traits and the immune infiltration subtypes of LUAD patients. The results revealed that the red module (containing 1,028 genes) and the light yellow module (containing 643 genes) showed a high correlation with the immune infiltration subtypes (r = 0.62, *P* = 9e-47; r = 0.69, *P* = 2e-60, respectively; **Figure 2B**). A high correlation between red modules and bright yellow modules was also revealed by the correlation cluster heatmap (**Figure 2C**). In addition, the high reliability of the WGCNA result was confirmed by the high correlation between GS and MM inside the modules (**Figures S2C, D**). The gene pairs with the Top 300 GS weighted coefficients in the two modules aforementioned were extracted, and the gene-related PPI map was constructed and plotted using the “Cytoscape” software. The PPI showed that the genes with the largest number of associated nodes in the weighted network of the two gene modules, such as NCKAP1L, CD53, SASH3, and CD3E, were present in the ssGSEA gene set (**Figures 2D, E**), and it revealed a strong correlation between the screened genes by WGCNA and the immune infiltration subtypes. The total 1,671 genes in the two modules were then analyzed by GO and KEGG pathway. The GO terms T cell activation, regulation of lymphocyte activation, external side of plasma membrane, and cytokine receptor binding were the enriched GO terms (**Figures 2F**, **S2E** and **S2G**). The cytokine−cytokine receptor interaction and chemokine signaling pathways were also enriched in the two modules as revealed by the KEGG pathway analysis (**Figures 2G**, **S2F** and **S2H**). These results confirmed that the genes in the two modules were immune related genes.

### Further Screening and Identification of Immune Infiltration Related Genes

**Figure 3:**
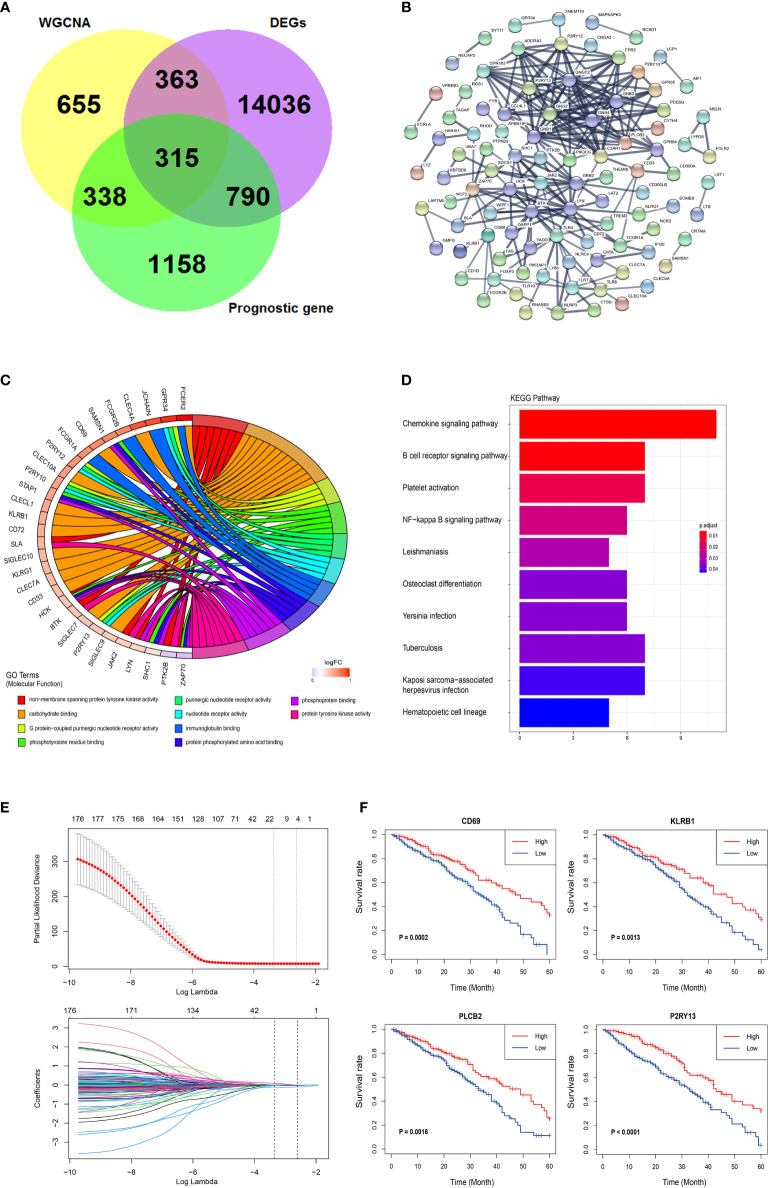
Further screening and identification of immune infiltration related genes. **(A)** Venn diagram showing the intersection (315 genes) of the genes related with immune infiltration screened by WGCNA and DEGs in different immune infiltration subtypes and the prognostic genes screened by log-rank test. **(B)** PPI network of the 178 genes. The line thickness indicates the strength of the data supporting the interaction. **(C)** A Chord graph showing the genes in the top 10 enriched GO terms in the molecular function level. **(D)** KEGG pathway analysis of the 178 genes. **(E)** Lasso-Cox regression showing the independent prognostic genes. The top graph shows the coefficient shrinkage, the bottom graph shows the 10-fold cross-validation. **(F)** The Kaplan-Meyer survival curves of the four genes (CD69, KLRB1, PLCB2, and P2RY13) screened by the Lasso-Cox regression.

DESeq2 standard procedure was used to screen DEGS in LUAD patients with high immune infiltration in comparison with patients with low or medium immune infiltration to further define the genes related to immune infiltration and their prognostic survival in LUAD patients (**Figures S3A, B**). In addition, a total of 2,601 genes related to the prognosis of LUAD were screened by univariate log-rank test. The coordinate expression genes associated to immune infiltration screened by WGCNA were intersected with DEGs and prognosis-related genes, and 325 genes were found as common genes (**Figure 3A**). The genes already included in ssGSEA analysis, non-coding genes (lincRNA and miRNA), pseudogenes, and antisense chains of coding genes were removed and a total of 178 genes remained that were used to construct a PPI network by the “STRING” website (which does not show single-node genes). In this network, some members of the G-protein coupled receptor pathway, such as GNG2, GNB2, and P2RY13, as the central nodes, had more associated genes, and the evidence of gene association is also more sufficient (thicker the lines, more sufficient the evidences) (**Figure 3B**). The GO analysis of the 178 genes revealed three molecular functional GO terms with the highest enrichment degree, such as non−membrane spanning protein tyrosine kinase activity, carbohydrate binding, and G protein−coupled purinergic nucleotide receptor activity (**Figure 3C**). The biological process and cellular component of the GO analysis revealed that immune response−activating related signaling pathways and cell membrane component were the mainly enriched GO terms (**Figures S3C, D**). The KEGG pathway analysis revealed that chemokine signaling pathway and B cell receptor pathway were the mainly enriched pathways (**Figure 3D**). Finally, the lasso-cox algorithm applied to these 178 genes revealed that CD69, KLRB1, PLCB2, and P2RY13 were independent prognostic genes (**Figure 3E**). These four genes showed specific interactions with various immune-related genes or immune-related signaling pathways as revealed by the PPI network (**Figures S3E–H**). The LUAD patients were divided into high expression group and low expression group according to the median expression value of these four key genes used as the cutoff value. The results showed that the 5-year survival rate in LUAD patients with high expression of these four genes was significantly higher than that in patients with low expression of these genes (**Figure 3F**).

### Relationship Between the Four Key Genes and LUAD Immune Microenvironment

**Figure 4:**
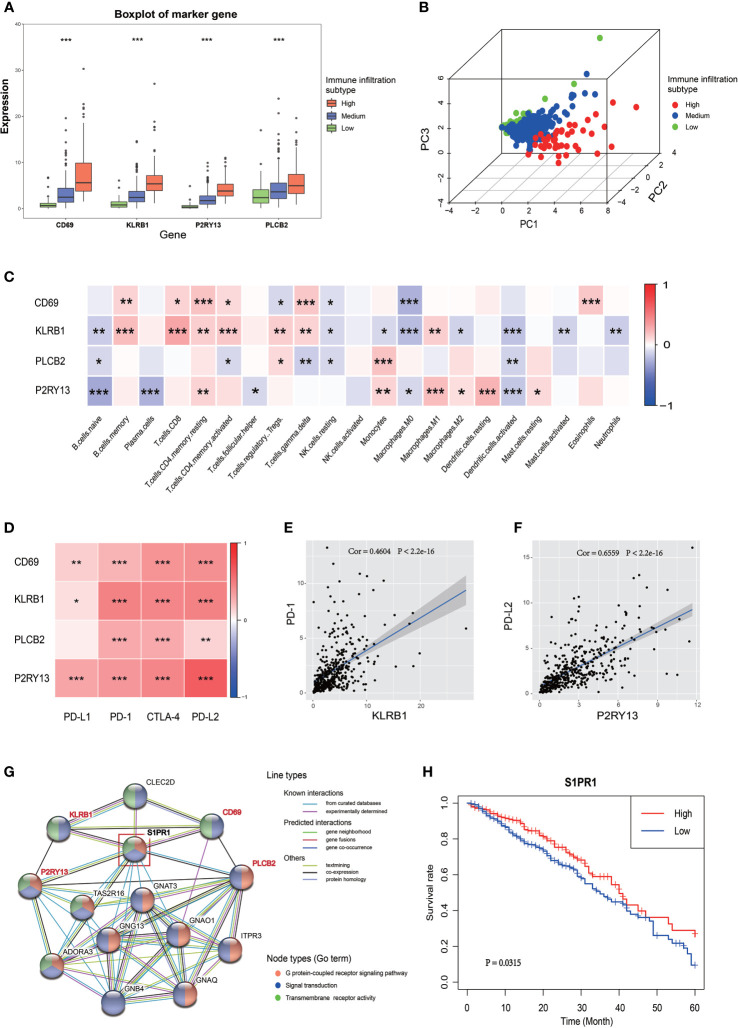
Relationship between the four key genes and LUAD immune microenvironment. **(A)** The boxplot shows the expression trends of the four key genes in different immune infiltration subtypes. **(B)** 3D PCA of the four key genes shows the spatial distribution of different immune infiltrations. **(C)** Correlation between the four key genes and 22 immune cell types. The blue box indicates a negative correlation, the red box indicates a positive correlation, and the increase of the color indicates the increase of the correlation coefficient. **(D)** Correlation between the four key genes and the four immune checkpoints. The increase of the color indicates the increase of the correlation coefficient. **(E)** Correlation between KLRB1 and PD-1. **(F)** Correlation between P2RY13 and PD-L2. **(G)** PPI network of the four key genes. The colors of the lines indicate the types of interaction, the colors of the node indicate which GO term the gene belong to. The node in the red box (S1PR1 gene) was the common interaction of the four key genes. **(H)** The Kaplan-Meyer survival curve of S1PR1 in LUAD. *P < 0.05; **P < 0.01; ***P < 0.001.

The expression of these four genes in different immune infiltration subgroups were analyzed to further explore the relationship between the four key genes and LUAD immune microenvironment and the potential mechanism regulating this relationship. A significant difference in the expression of these genes among the three immune infiltration subgroups was found (**Figure 4A**), all showing a progressive increase in their expression from the low immune infiltration subgroup to the high one. The PCA suggested that different immune infiltration states could be distinguished to some extent just only using the expression of these four key genes (**Figure 4B**). A correlation between the expression of these key genes and the infiltration of various immune cells was indeed found (**Figures 4C** and **S4A**). CD69 is a marker of macrophage activation and, as such, is negatively correlated with the relative number of M0 cell. KLRB1 expression was positively correlated with the activation of CD8 positive T cells, PLCB2 expression was positively correlated with monocyte infiltration, and P2RY13 expression was negatively correlated with the number of naive B cell, but positively correlated with the number of resting dendritic cells. A correlation between the expression of the four key genes and immune checkpoint genes was also found, as shown in **Figures 4D** and **S4B–J**, in which the correlation between KLRB1 and PD-1, as well as P2RY13 and PD-L2 was significant (**Figures 4E, F**). The potential interaction between these four key genes was also evaluated. The use of the STRING website revealed that both P2RY13 and PLCB2 belong to the G-protein coupled receptor pathway, while CD69 and KLRB1 belong to the signal transduction pathway, but these four genes had a common interaction gene, such as S1PR1, which is a member of both the G-protein coupled receptor pathway and the signal transduction pathway (**Figure 4G**). The subsequent investigation of S1PR1 revealed that it might also be a prognostic gene in LUAD (in TCGA cohort and GSE72094), with interactions with some well-known tumor genes such as AKT1, STAT3 and CXCR4 (**Figures 4H**, **S4K–4N**).

### Establishment of a Prognostic Model Based on Four Immune-Related Genes

**Figure 5:**
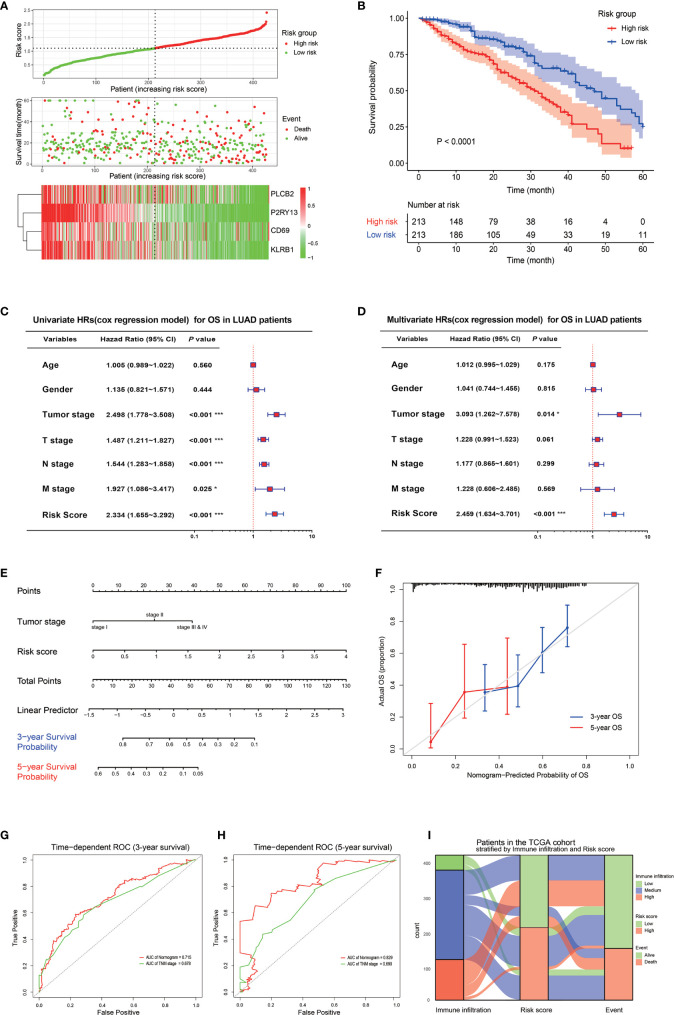
Prognostic value of the four key genes. **(A)** Risk score signature based on the four key genes. The top graph shows the calculation formula and the value of the risk score; the middle graph shows the distribution of the survival status based on the risk score; the bottom graph shows the cluster heatmap of the four key genes. **(B)** The Kaplan-Meyer survival curve shows the different survival rate between LUAD patients with high-risk score and low-risk score. **(C)** The forest plot shows the result of the univariate cox regression for the overall survival in TCGA cohort. **(D)** The forest plot shows the result of the multivariate cox regression for the overall survival in the TCGA cohort. **(E)** Nomogram based on the multivariate cox regression for the prediction of the 3-year and 5-year survival rates in the TCGA cohort. **(F)** An internal calibration curve shows the fitness between the actual overall survival probability and the nomogram-predicted overall survival probability. **(G, H)** The time-dependent ROCs show the accuracy of the Nomogram and TNM for the 3-year overall survival prediction **(G)** and 5-year overall survival prediction **(H)**. **(I)** The Sankey diagram shows the final survival status of LUAD patients with different immune infiltrations and with different risk scores. *P < 0.05; **P < 0.01; ***P < 0.001.

A risk score signature was constructed to integrate the roles of these four key genes by the coefficients of LASSO-Cox regression in view of their role in the prognosis and immune infiltration in LUAD (**Figure 5A**). The coefficients are shown in **Table S1**. The LUAD patients were divided into high-risk group and low-risk group according to the median value of the risk score. The survival rate of the patients in the low-risk group was significantly higher than that of the patients in the high-risk group, with a 5-year survival rate of 46.0 and 29.0%, respectively (**Figure 5B**). The combination of the risk score with the clinical baseline index of LUAD by univariate COX analysis revealed that tumor stage, TNM stage, and risk score were prognosis predictors, but after the correction by multivariate COX, only the tumor stage and risk score resulted as independent predictors of LUAD prognosis (**Figures 5C, D**). The result of the multivariate COX model was then quantified and visualized through the construction of a Nomogram to predict the 3-year and 5-year survival rates of LUAD patients (**Figure 5E**). A high coincidence rate between the predicted probability and the actual probability in the 3-year and 5-year survival prediction of LUAD was found through the internal calibration curves (**Figure 5F**). The time-dependent ROC curve showed that the area under the curve (AUC) of the Nomogram to predict the 3-year survival rate was 0.715, which was higher than that of the TNM stage (0.618), while the AUC to predict the 5-year survival rate was 0.829, also higher than 0.699 of the TNM (**Figures 5G, H**). The Sankey diagram visualized the relationship between the risk score and the final outcome of patients with different immune infiltration subtypes (**Figure 5I**).

### Validation of the Prognostic Value of the Four Immune-Related Genes in GEO Datasets

**Figure 6:**
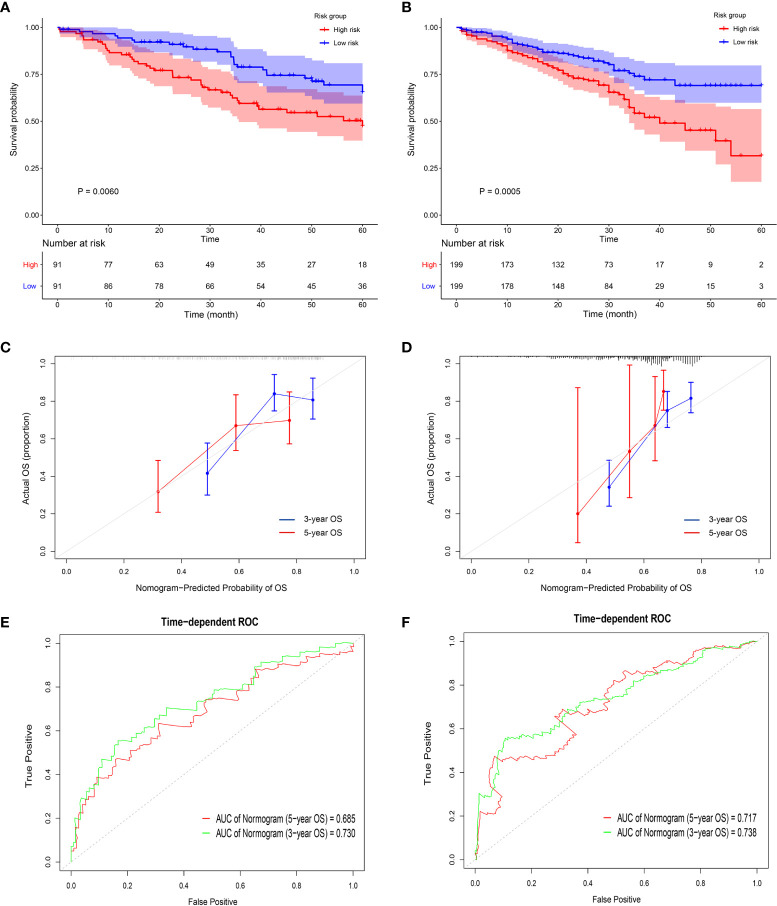
Validation of the prognostic value of the four key genes in the GEO datasets. **(A, B)** The Kaplan-Meyer survival curves show the different survival rate between high-risk group and low-risk group in GSE 41271 **(A)** and GSE 72094 **(B)**. **(C, D)** The external calibration curves show the fitness of the overall survival probability in GSE 41271 **(C)** and in GSE 72094 **(D)**. **(E, F)** The time-dependent ROCs show the accuracy of the 3-year overall survival prediction and 5-year overall survival prediction in GSE 41271 **(E)** and in GSE 72094 **(F)**.

Universality is an important index to evaluate a prognostic model, thus, it is necessary to use data from different sources to externally verify the Nomogram (33). Five GEO datasets using different chip platforms were selected to confirm the prognostic prediction ability of the immune-related genes and the Nomogram. All the four key genes had a prognostic significance in both GSE41271 and GSE72094 (**Figures S5A, B**). As regard the other GEO datasets, CD69 and KLRB1 in GSE50081, KLRB1 and PLCB2 in GSE68465, and PLCB2 and P2RY13 in GSE42127 also had a prognostic significance. The risk score constructed by the four key genes in GSE41271 and GSE72094 had a significant prognostic value (**Figures 6A, B** and **S5C, D**). The external calibration curves of the Nomogram to predict the two GEO datasets showed that the prediction of the 3-year and 5-year survival rate was in good agreement with the actual survival rates (**Figures 6C, D**). The ROC curves also revealed that the predicted AUCs of other survival periods were higher than 0.7, except for the AUC of the 5-year survival in GSE41271 (**Figures 6E, F**).

## Discussion

The continuous development of high-throughput sequencing technology allowed a deeper understanding of the genetic and epigenetic pathological characteristics of tumors, including LUAD. A variety of clustering and deconvolution algorithms are used to determine the state of TME (especially the immune TME) through the high-throughput RNA sequencing data, resulting in a great improvement of tumor treatment and prognosis (34). Moreover and more importantly, the potential association of the changes in tumor immune microenvironment with the gene expression changes may be helpful in finding the key genes leading to tumor immune infiltration. These key genes may represent novel biomarkers to predict the clinical outcome of patients or potential immunotherapeutic targets to develop effective anti-tumor drugs. However, most of the studies available based on bioinformatics only focus on algorithms to screen DEGs, lacking the in-depth investigation of the interaction network among these key genes, or single-source data creating an overfitting model with a weak universal survival prediction.

In this work the tumor immune status of LUAD patients in the TCGA database at the genetic level was considered. Based on ssGSEA algorithm, LUAD patients were divided into high, medium, and low immune infiltration subtype, and the Kaplan-Meyer analysis revealed a better prognosis of the patients with high immune infiltration subtype. These results suggested that a high immune infiltration specifically localized in the tumor might be considered as an anti-tumor factor. The expression of genes implicated in immunotherapy and specific genes of immune cells, along with the abundance of immune cell infiltrates in a tumor, is substantially inversely correlated with tumor purity (35). This aspect underlines the need to consider tumor purity when evaluating the gene expression of markers obtained from tumor transcriptome data. The ESTIMATE algorithm was used to calculate the tumor purity and the immune and stromal score in LUAD, thus, the negative regulation relationship between the immune infiltration state and tumor burden in LUAD was confirmed. Unlike the ssGSEA, CIBERSORT deconvolution algorithm, only focus on the proportion of immune cells in tumors. The CIBERSORT results showed that mainly CD8+ cells, activated CD4 memory cells and M1 macrophages are the ones highly infiltrated in a tumor in a condition of high immune infiltration, and these cells are important anti-tumor immune cells (36).

Whether it is tumor immune cells or tumor immune-related genes, there is often a network-based synergistic relationship between them often exists (37–39). If the immune infiltration state of LUAD is classified and confirmed, it is important to evaluate which are the coordinate expression genes, and the correlation between them and the immune infiltration in LUAD. For this reason, the immune infiltration of LUAD in this work was considered as a clinical trait, and the WGCNA algorithm was used to screen the coordinate expressed genes in the form of gene modules that were associated with the immune infiltration of LUAD. A total of 1,671 genes included in two gene modules were screened and the PPI network analysis showed that the hub gene in the two modules were included in the gene set of ssGSEA algorithm. GO and KEGG analysis also confirmed that these genes were mainly enriched in immune-related pathways. These results suggested that the WGCNA method used in our study to screen immune-related genes was effective and reliable.

A further cleaning of the aforementioned 1,671 genes resulted in the selection of 178 genes and their PPI network was constructed by “STRING” website. In this network, some hub genes such as GNB2, GNB4, GNG2, GNGT2, and P2RY13 had more interactional genes and more sufficient evidences of the interactions. These hub genes are all members of the G protein-coupled receptor pathway. The GO enriched analysis revealed that the G protein-coupled purinergic nucleotide receptor activity pathway also ranked third in the molecular function Go terms. The G protein-coupled receptor pathway is nowadays a hot spot in cancer immune research, and some members of GPCRs are actually demonstrated as having a role as prognostic factors in a variety of cancers (40–42).

The LASSO algorithm was used to find independent prognostic molecules and avoid co-linearity between genes since the genes screened by WGCNA are often coordinate expression genes, and four key genes such as CD69, KLRB1, PLCB2, and P2RY13 were finally found. The relationship between CD69 and tumor immunity is known, although it was initially considered as a marker of early activation of T cells and macrophages, but the latest research revealed that it is a surface marker of tissue resident memory T cells, and high infiltration of these cells often indicates a better tumor outcome (43, 44). KLRB1 (also called CD161) is a gene encoding for a surface marker of many subtypes of T cells and NK cells, and its widespread expression is associated with a better prognosis of NSCLC (45, 46). PLCB2 and P2RY13 are members of the phosphatidyl C family and purine subunit family, respectively (47, 48). These two genes are classified as belonging to the GPCRs pathways that are closely related to tumor immunity and despite reports on the relationship between these two genes and tumor immunity are still rare, new progress has been made revealing their relationship with macrophages and NK cells (49, 50). In this study, the expression of these four genes was increased with the increase of LUAD immune infiltration level. The important aspect was that these four genes were correlated with the amount of immune cell infiltration (such as CD69 and M0 macrophage), but also with the expression of immune checkpoint genes (such as KLRB1 and PD-1, P2RY13, and PD-L2) suggesting that these four genes might predict the immunotherapy response and could be potentially considered as new immunotherapeutic targets. These four genes interact with a gene common to all of them, such as S1PR1, which is a core gene of the G protein coupled pathway (51). S1PR1 is usually considered as an oncogene, since it promotes the proliferation, invasion, and metastasis of tumor cells through the STAT3, PI3K/AKT, and CCR signaling pathway in many types of malignant tumors (52–54). However, some recent studies revealed that S1PR1 is also able to promote tumor cell apoptosis, thus, its high expression is an indicator of a good prognosis (55–57). In the present study, our hypothesis was that an interacting gene network was formed among CD69, KLRB1 and GPRCs family members PLCB2, P2RY13, and S1PR1. This gene network further activated the downstream genes in GPRCs signaling pathway, promoting the immune infiltration in LUAD tissues and the consequent anti-tumor effect of immune cells. This finding provided a new basis clarifying the mechanism of immune infiltration in LUAD, and providing a potential therapeutic target for the immunotherapy against LUAD.

Finally, these four genes were used to construct a risk score signature in training cohort to explore the role of these four genes in the clinical prognosis of LUAD. This risk score signature allowed the identification of high-risk LUAD patients with poor prognosis. This score signature was also used as an independent prognostic index to construct an effective Nomogram prediction model combined with the tumor stage in LUAD TCGA cohort. This model could predict the 3-year and 5-year survival rates of TCGA LUAD with a high accuracy. The ROC curves revealed that the AUC of the Nomogram were better than AUC of the TNM stage. Tumor heterogeneity is an unavoidable problem, thus, it should be considered in all tumor studies, being also a problem in the prognostic prediction of LUAD patients (58, 59). The model was externally validated in two GEO datasets using different microarray platforms to further improve the universality and robustness of the Nomogram in LUAD samples. The validation further confirmed the prognostic value of these four genes and the ability of the Nomogram to predict the survival rates in LUAD patients. These results indicated that these four LUAD genes had a universal prognostic value, thus, they might be potentially considered for further clinical applications.

Our study had several limitations that should be acknowledged while indicating necessary future studies in the relevant areas. As shown in the results, the prognostic significance of the four genes and S1PR1 were found in some of the examined GEO datasets, not all. It may be due to the limitations of the data obtained from open datasets. The data were shared by studies using inconsistent experimental study design, such as different detected objects, various detected platforms, and diverse sample sizes. For example, the GSE50081 only focused on early-stage NSCLC, and the GSE41271 and the GSE42127 did not contain S1PR1 detection probes. The heterogeneity of tumors, which was also hard to control using open data, may have impacted and resulted in these inconsistent findings among datasets as well. Specifically, the individual differences, difference of tumor development stages, and difference of tumor sites could all affect the analysis results of immune cell infiltration and gene expression levels. All these factors can be impactful to the data analysis, lower the validation of the prognostic value of these genes, and thus affect the universality of the prognostic prediction model. For future studies, the clinical application value of this gene signature needs to be further verified in more independent LUAD cohorts. Our research team has just launched a new validation study at the protein level based on an independent cohort of which the samples are being collected from our own hospital. Meanwhile, a larger sample size should be considered in future studies to help reduce the interference caused by tumor heterogeneity and ensure statistic power.

## Conclusions

Our study identified four key genes significantly correlated with tumor immune infiltration and in LUAD and its prognosis. These four genes formed a network with S1PR1, which is a mutual interaction gene, activating the downstream genes in GPRCs to promote the immune infiltration in LUAD. The constructed risk score signature based on the key genes could be used as an independent biomarker to predict the prognosis of LUAD. Therefore, the screening, identification, and analysis of these four genes made a contribution in the understanding of GPCRs and the immune infiltration in LUAD, opening up new perspectives for more effective immunotherapeutic strategies.

## Data Availability Statement

The datasets presented in this study can be found in online repositories. The names of the repository/repositories and accession number(s) can be found in the article/**Supplementary Material**.

## Author Contributions

YW and FX conceived, designed, and wrote the manuscript. LQ and PY analyzed the data. YC and XZ helped with manuscript and data review. All authors contributed to the article and approved the submitted version.

## Funding

This study was funded by the National Natural Science Foundation of China (Grant No. 81701213), the Natural Science Foundation of Jiangsu Province of China (Grant No. BK20170369) and Science and Technology Project of Nantong (Grant No. JCZ20209).

## Conflict of Interest

The authors declare that the research was conducted in the absence of any commercial or financial relationships that could be construed as a potential conflict of interest.

## Abbreviations

AUC, Area Under the Curve; BP, Biological Process; CC, Cellular Component; CCR, Cell Surface Chemokine Receptor; CD, Cluster of Differentiation; CTLA-4, Cytotoxic T Lymphocyte associated Antigen 4; DCs, Dendritic Cells; DEGs, Differential Expressed Genes; FPKM, Fragments Per Kilobase Million; GEO, Gene Expression Omnibus; GPCRs, G-Protein Coupled Receptors; GS, Gene Significance; HLA, Human Leukocyte Antigen; HR, Hazard Ratio; IFN, Interferon; KEGG, Kyoto Encyclopedia of Genes and Genomes; KLRB1, Killer Cell Lectin Like Receptor B1; LASSO, Least Absolute Shrinkage and Selection Operator; LUAD, Lung Adenocarcinoma; MEs, Module Eigengenes; MF, Molecular Function; MHC, Major Histocompatibility Complex; MM, Module Membership; NK, Natural Killer; NSCLC, Non-Small Cell Lung Cancer; OS, Overall Survival; PCA, Principal Component Analysis; PD-1, Programmed cell Death 1; PD-L1, Programmed cell Death Ligand 1; PLCB2, Phospholipase C Beta 2; PPI, Protein-Protein Interaction; P2RY13, Purinergic Receptor P2Y13; ROC, Receiver Operating Characteristic curve; SCLC, Small Cell Lung Cancer; ssGSEA, single sample Gene Set Enrichment Analysis; S1PR1, Sphingosine-1-Phosphate Receptor 1; TCGA, The Cancer Genome Atlas; TIL, Tumor Infiltrating Lymphocyte; TME, Tumor Microenvironment; WGCNA, Weighted Correlation Network Analysis.
