# Supplementary material for: Screening and Identification of Four Prognostic Genes Related to Immune Infiltration and G-Protein Coupled Receptors Pathway in Lung Adenocarcinoma
Source: Front Oncol. 2021 Feb 8;10:622251. doi: 10.3389/fonc.2020.622251 (PMC7897677; doi:10.3389/fonc.2020.622251)
Supplement: Supplementary file 1 [file DataSheet_1.zip › Supplementary materials/Supplementary Materials.docx]

**Supplementary Materials**

**Supplementary Tables**

**Table S1. Coefficients of the 4 key genes in TCGA cohort**

| Gene names | Coefficients |
| --- | --- |
| CD69 | - 0.03437 |
| KLRB1 | - 0.06325 |
| PLCB2 | - 0.04340 |
| P2RY13 | - 0.11558 |

**Supplementary Figure Legends**

**Figure S1. Identification immune infiltration subtypes of LUAD in TCGA cohort.** (A) According to 29 immune-related gene sets, 426 samples were clustered in 3 immune infiltration subtypes by K-means method. (B, C & D) Tumor purity (B), ESTIMATE score (C) and stromal score (D) were calculated by ESTIMATE algorithm in different immune infiltration subtypes. (E) Relative expression levels of 22 immune cells types in 426 LUAD samples.

* : P < 0.05; **: P < 0.01; ***: P < 0.001

**Figure S2. Detection of immune infiltration related modules and genes by WGCNA.** (A & B) Selection of the appropriate soft threshold for WGCNA. The soft threshold based on scale independence (A), the soft threshold based on mean connectivity (B). (C & D) The correlation plots between module membership and gene significance in red module (C) and in lightyellow module (D). (E & F) The GO enriched analysis (E) and KEGG pathway analysis (F) of genes in red module. (G & H) The GO enriched analysis (G) and KEGG pathway analysis (H) of genes in lightyellow module.

**Figure S3. Further screening and identification of immune infiltration related genes.** (A) A volcanoplot showed the DEGs in different immune infiltration subtypes (B) The heatmap showed the top 300 DEGs with biggest absolute value of Padj in 426 LUAD samples. (C & D) The Chord graph showed the genes in top 10 enriched GO terms in biological process (C) and cellular component (D). (E-H) PPI networks of the 4 key genes.

**Figure S4. Relationship between the 4 key genes and LUAD immune microenvironment.** (A) The correlation between the 4 key genes and 22 immune cell types. The correlation coefficients were showed in the box. (B-J) The correlations between 4 key genes and 4 immune checkpoint genes. (K) The expression levels of S1PR1 in different immune infiltration subtypes. (L) PPI network of the S1PR1. The colors of line indicate the types of interaction evidence, the colors of node indicate which GO term the gene belong to. (M & N) The K-M survival curves of the LUAD patients with different SIPR1 expression levels in GSE72094 and GSE50081.

* : P < 0.05; **: P < 0.01; ***: P < 0.001

**Figure S5. Validation of the prognostic value of 4 key genes in GEO datasets.** (A & B) The K-M survival curves of LUAD patients with different 4 key genes expression levels in GSE 41271 (A) and GSE 72094 (B). (C & D) The risk score signature based on the 4 key genes in GSE 41271 (C) and GSE 72094 (D). Top graphs showed the calculation formula and the value of risk score; middle graphs showed the distribution of the survival status based on the risk score; bottom graphs showed the cluster heatmap of the 4 key genes.
