# Supplementary figures and images for: Screening and Identification of Four Prognostic Genes Related to Immune Infiltration and G-Protein Coupled Receptors Pathway in Lung Adenocarcinoma
Source: Front Oncol. 2021 Feb 8;10:622251. doi: 10.3389/fonc.2020.622251 (PMC7897677; doi:10.3389/fonc.2020.622251)

A

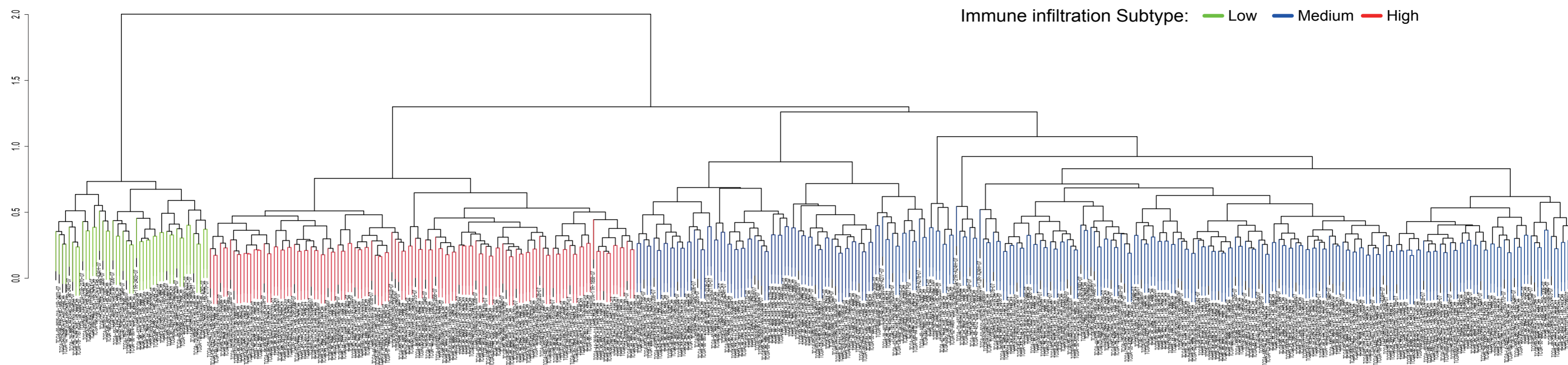

B

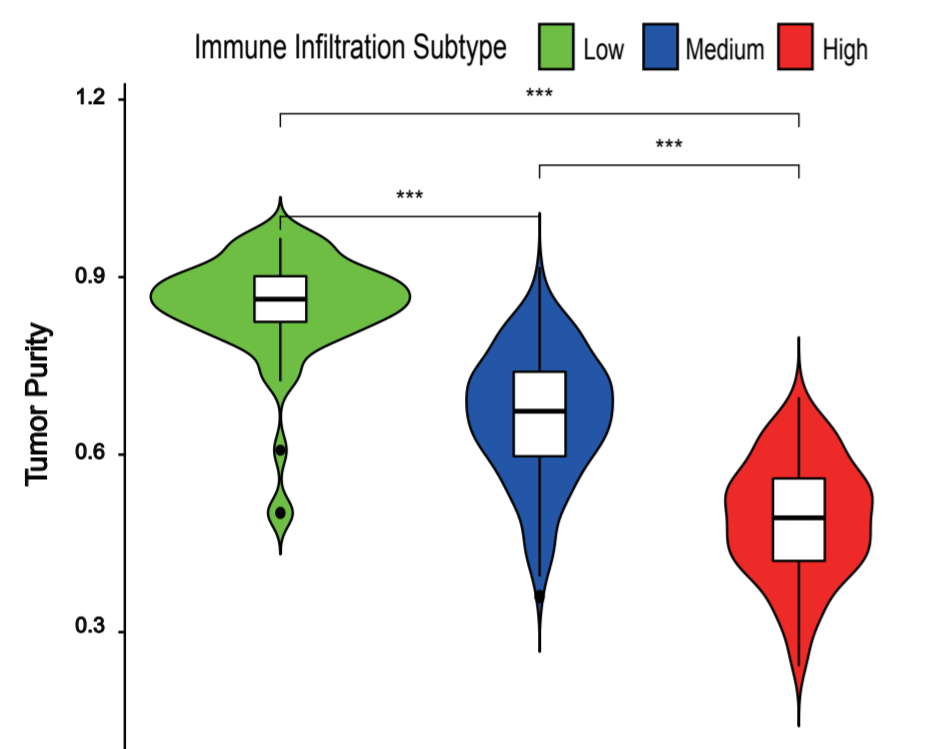

C

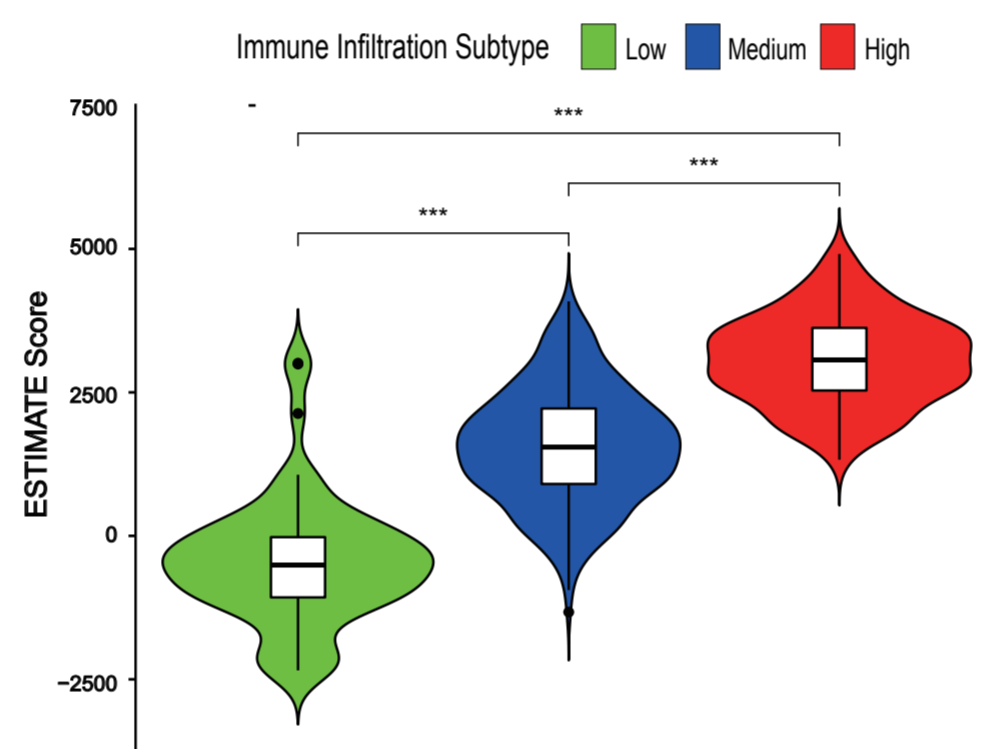

D

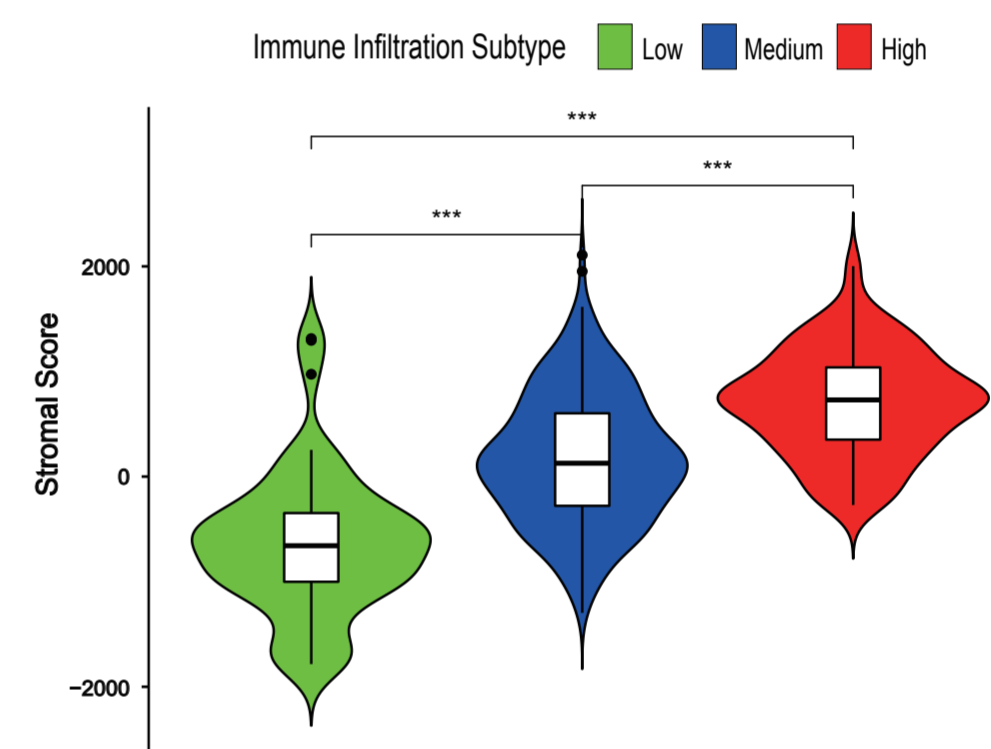

E

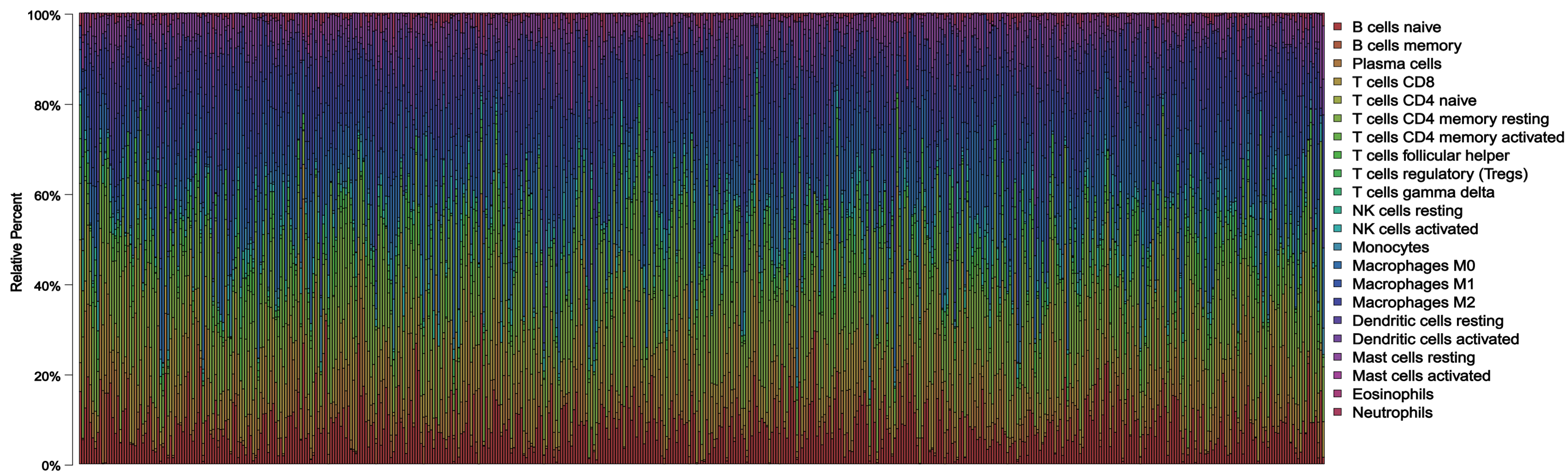

Supplement: Supplementary file 1 [file DataSheet_1.zip › Supplementary materials/Supplementary Figures/Figure-S1.pdf]

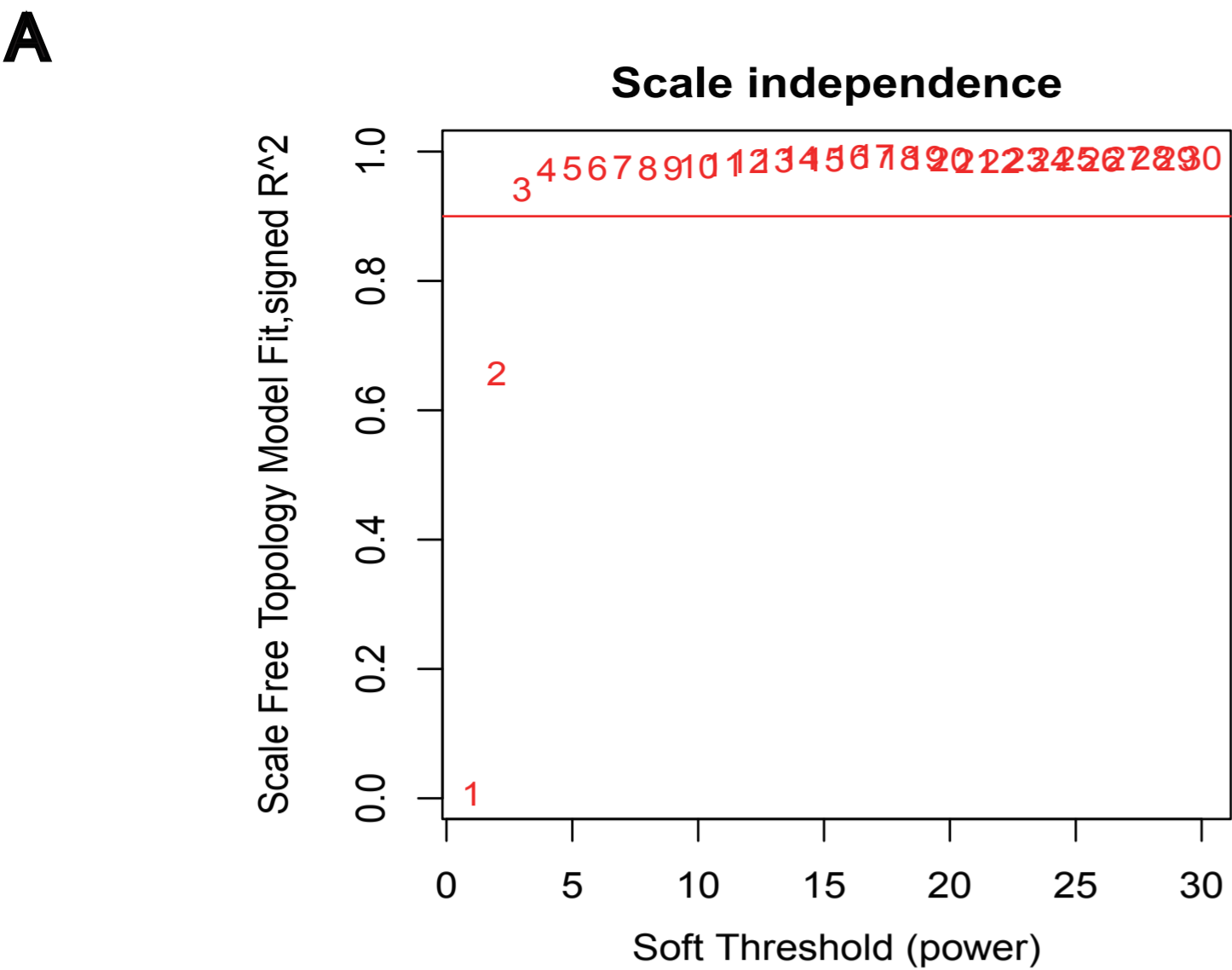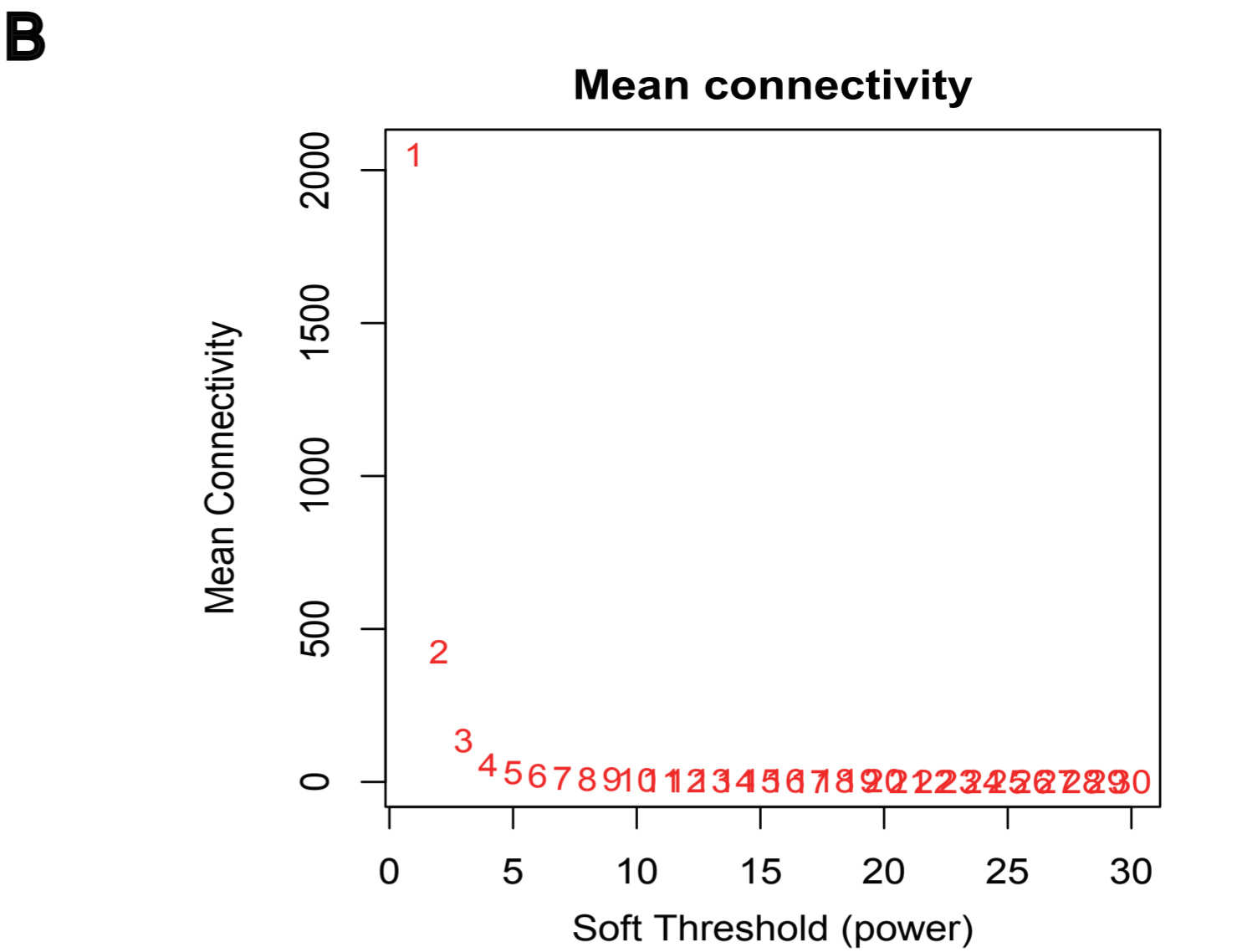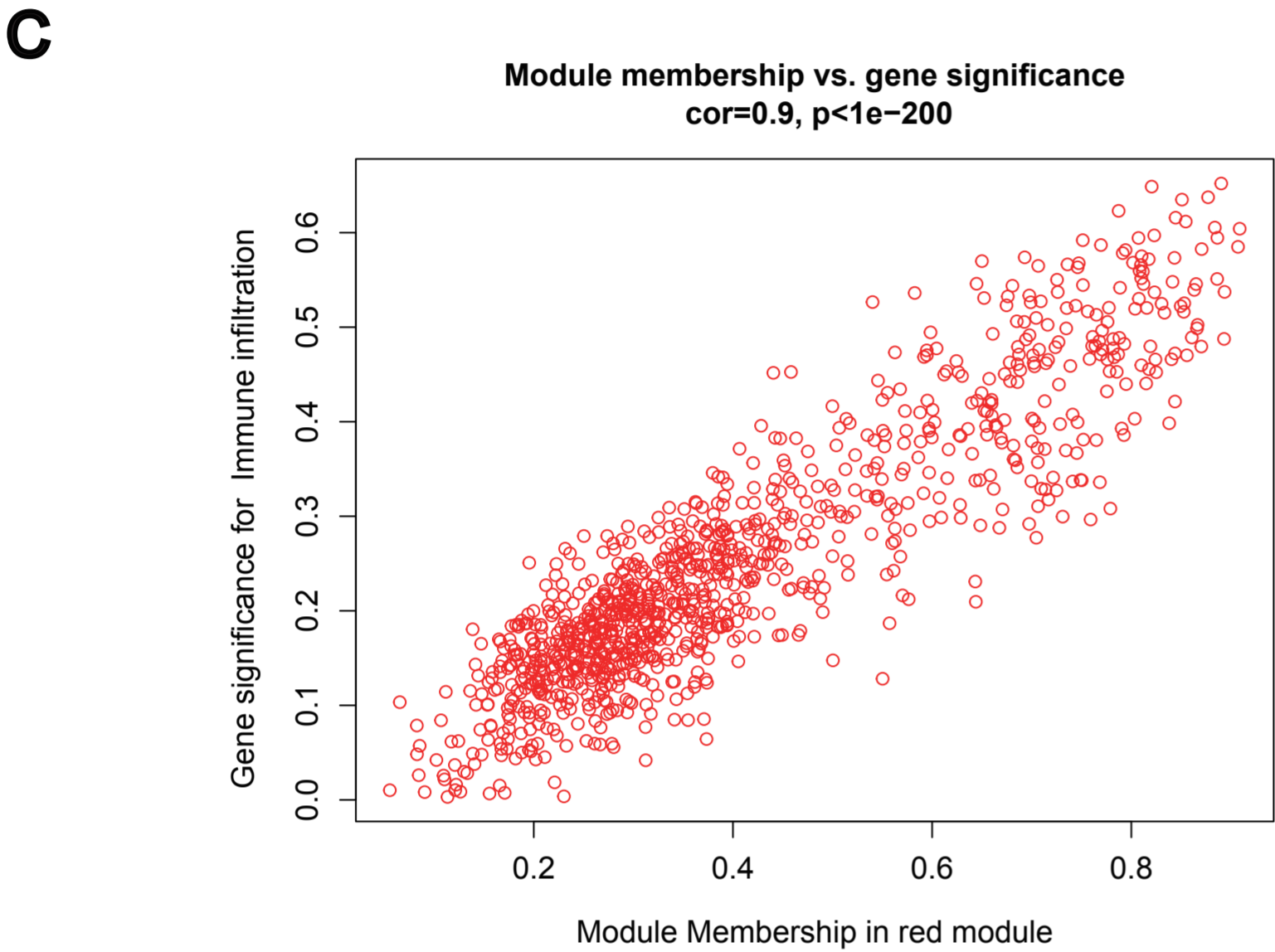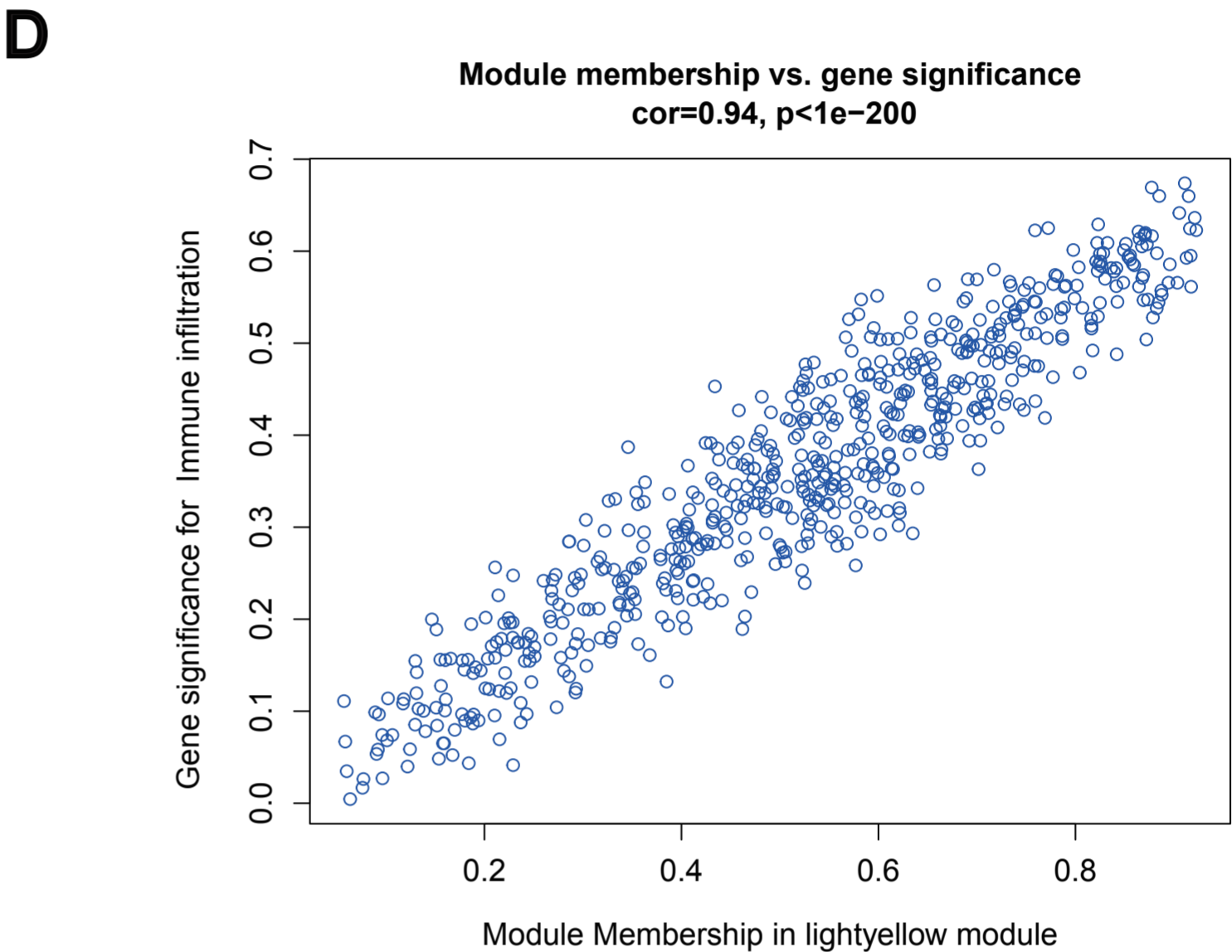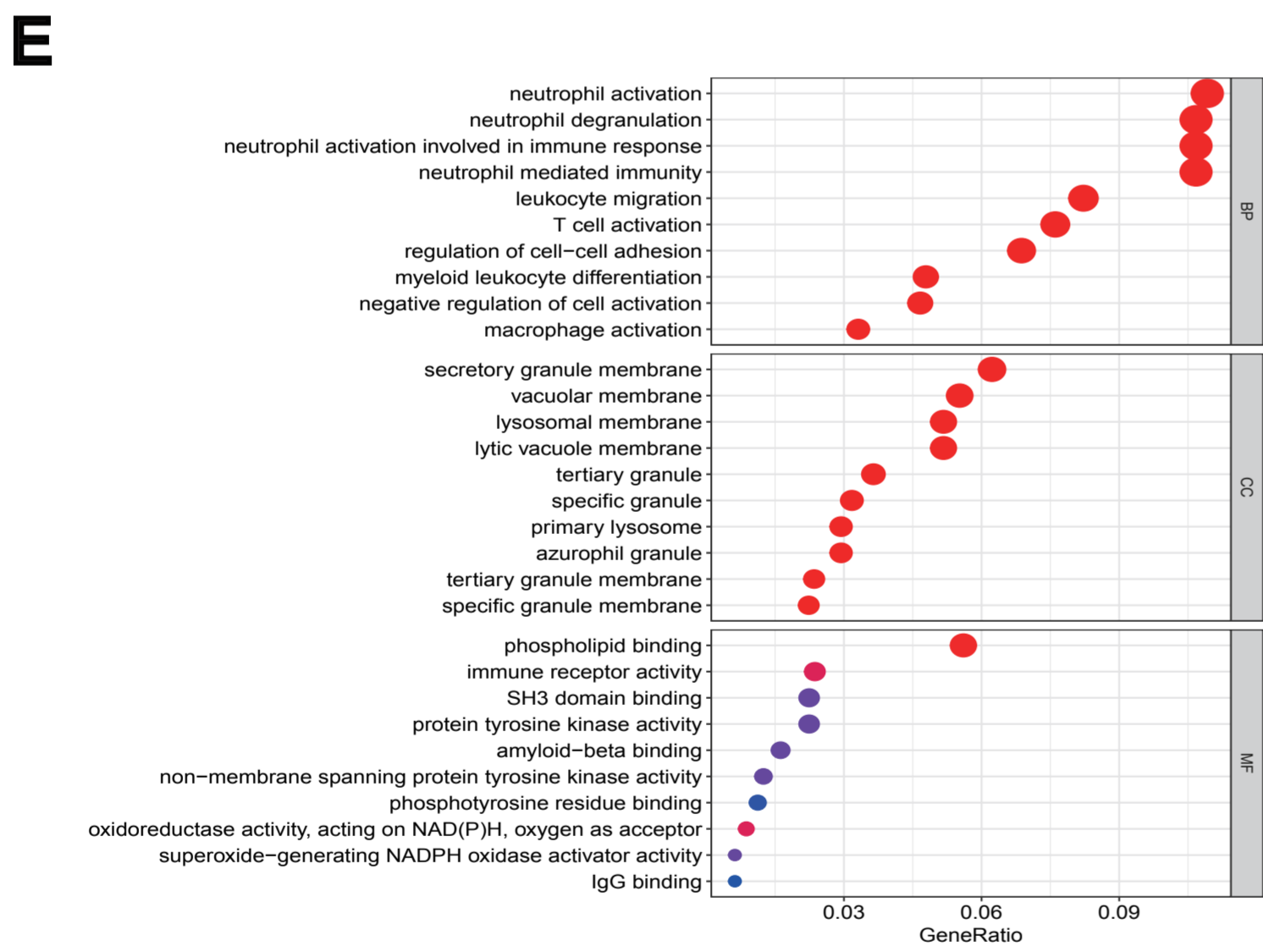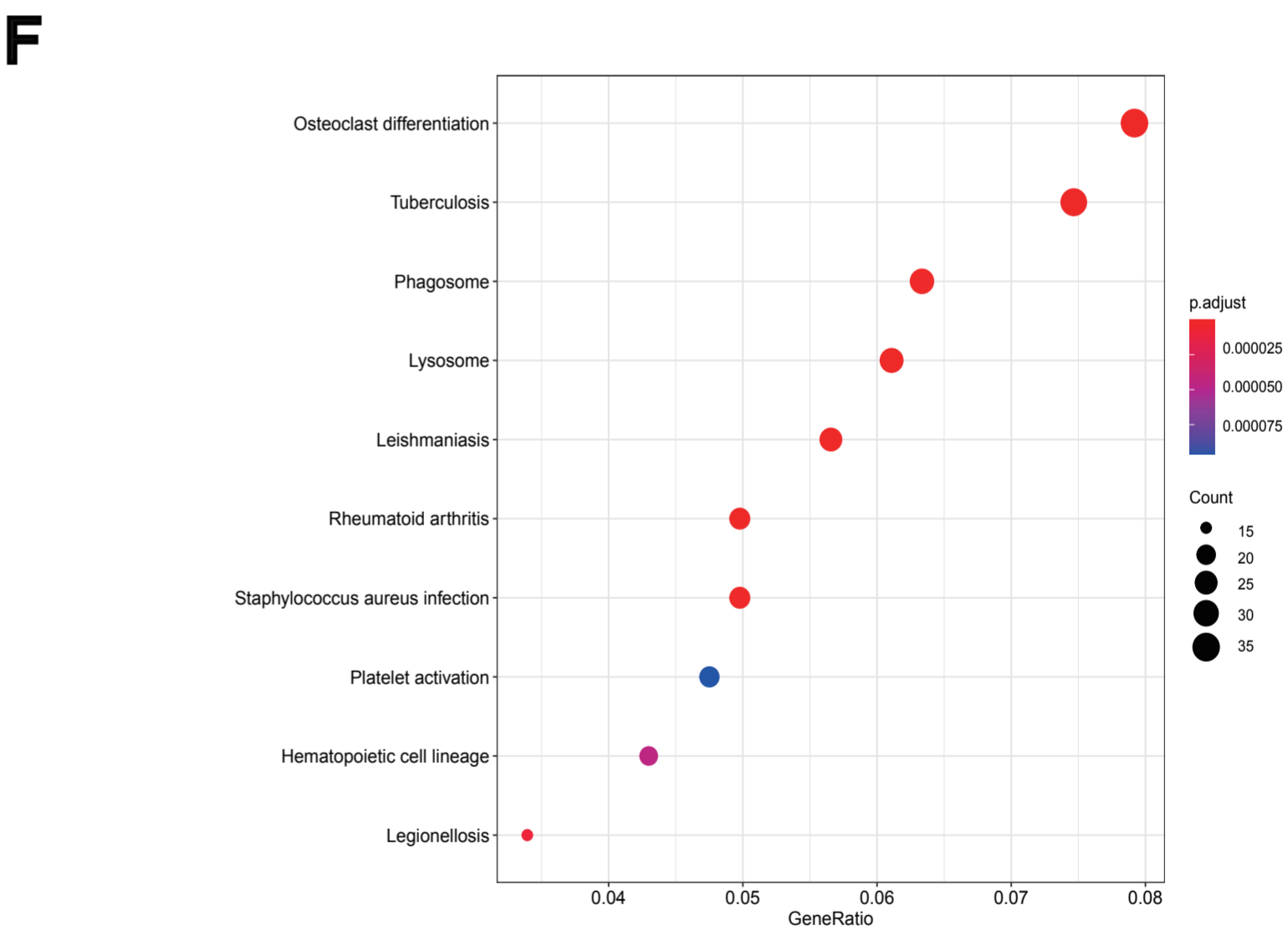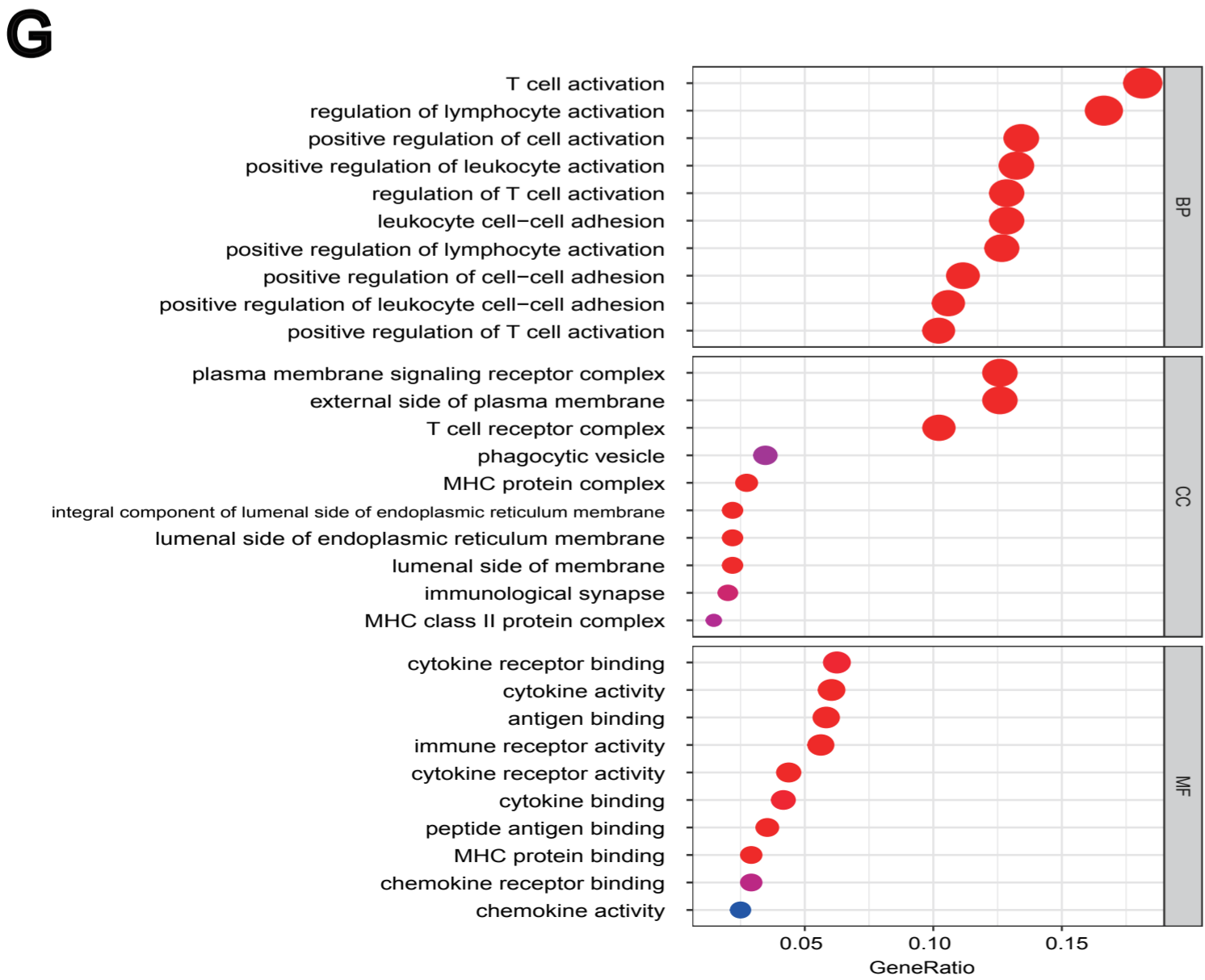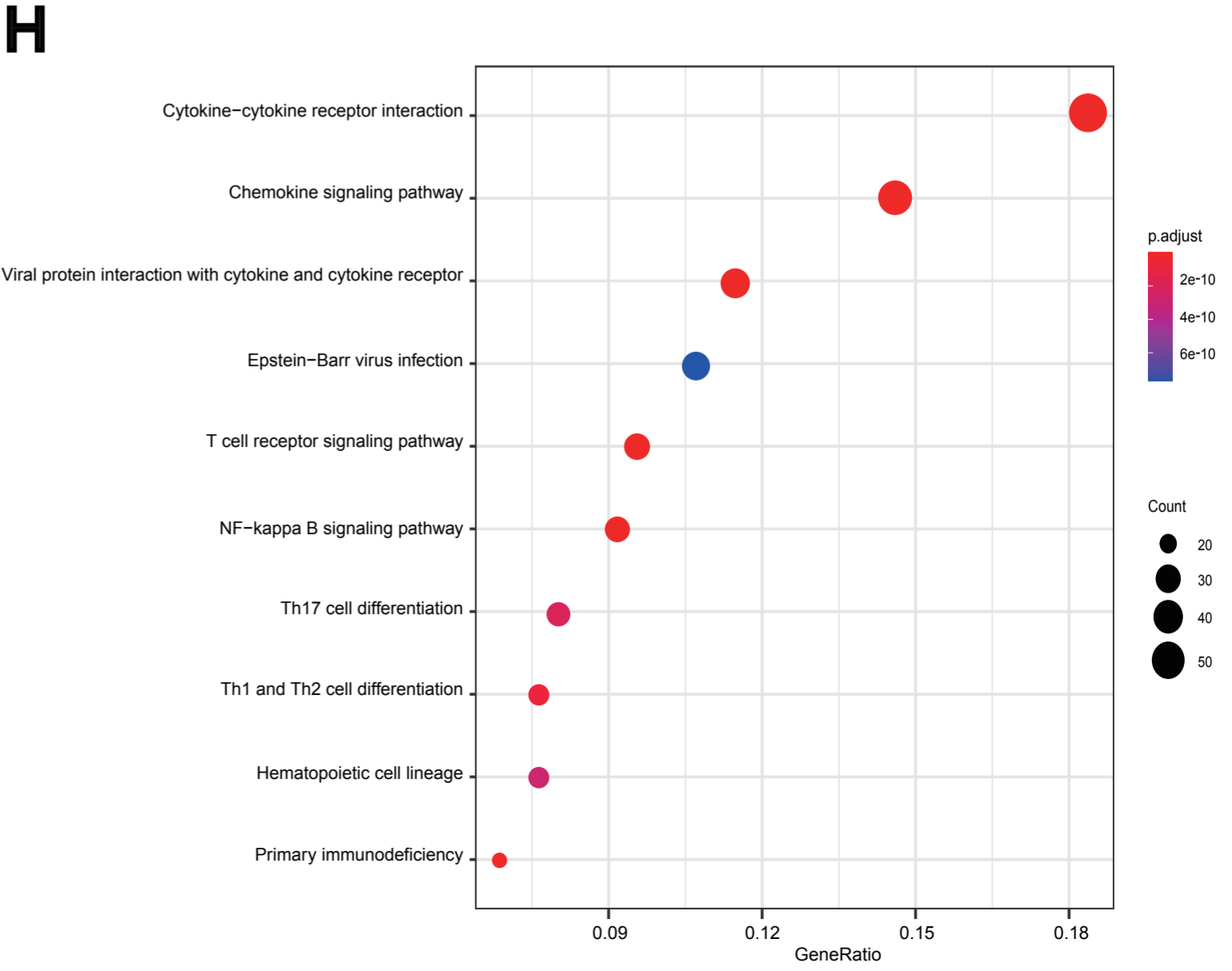

Supplement: Supplementary file 1 [file DataSheet_1.zip › Supplementary materials/Supplementary Figures/Figure-S2.pdf]

A

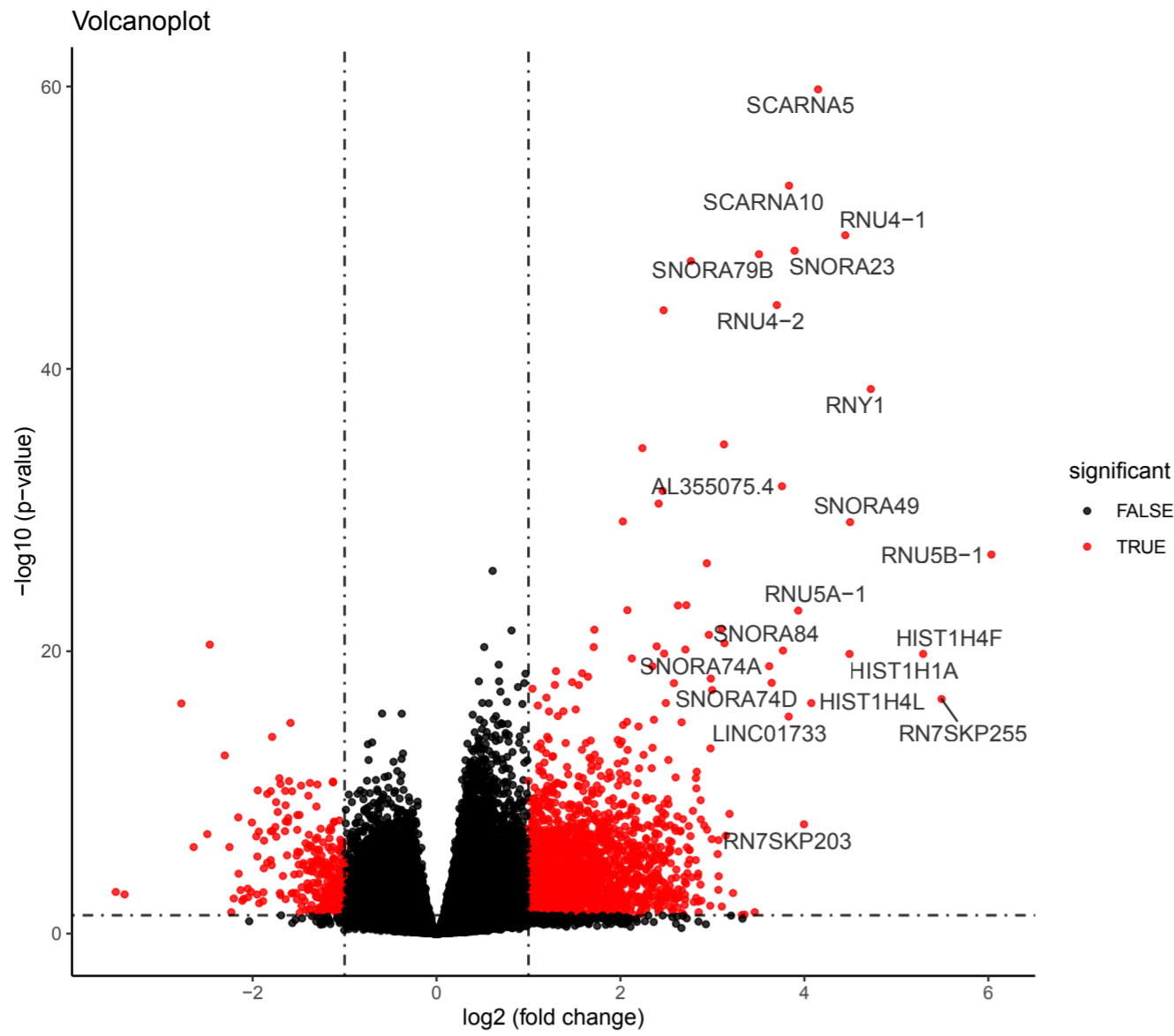

B

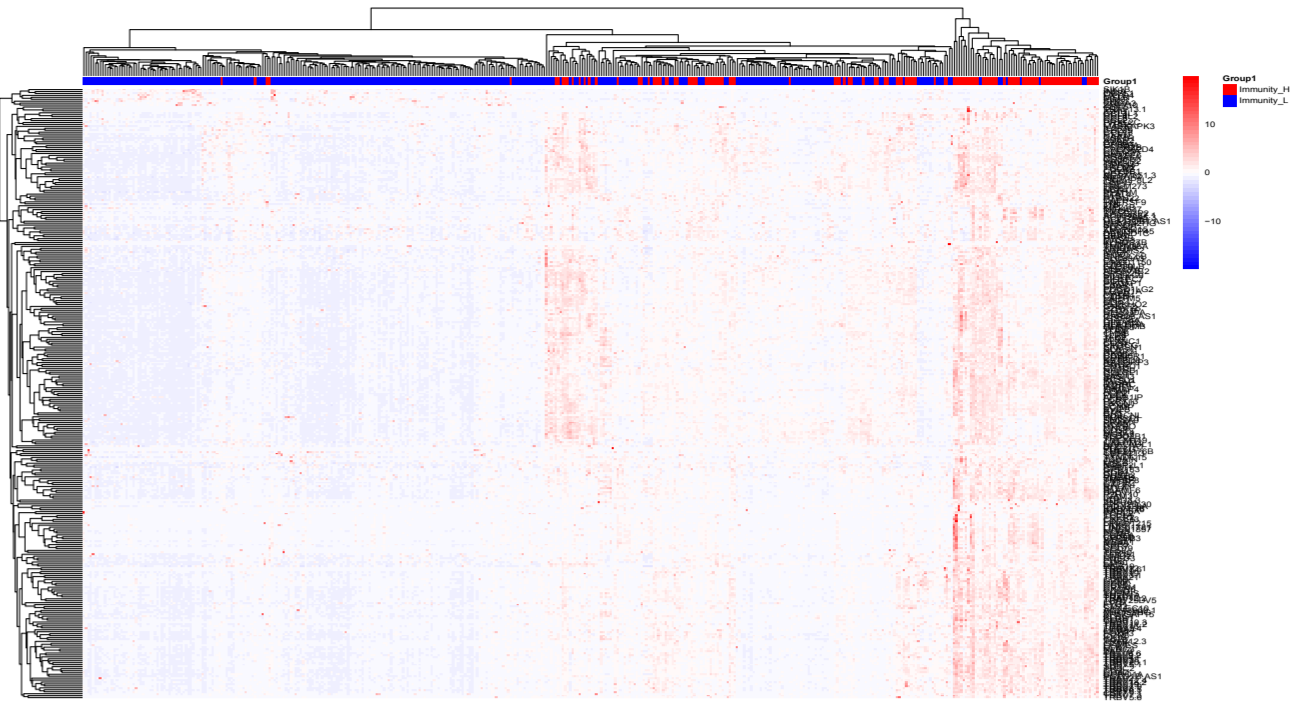

C

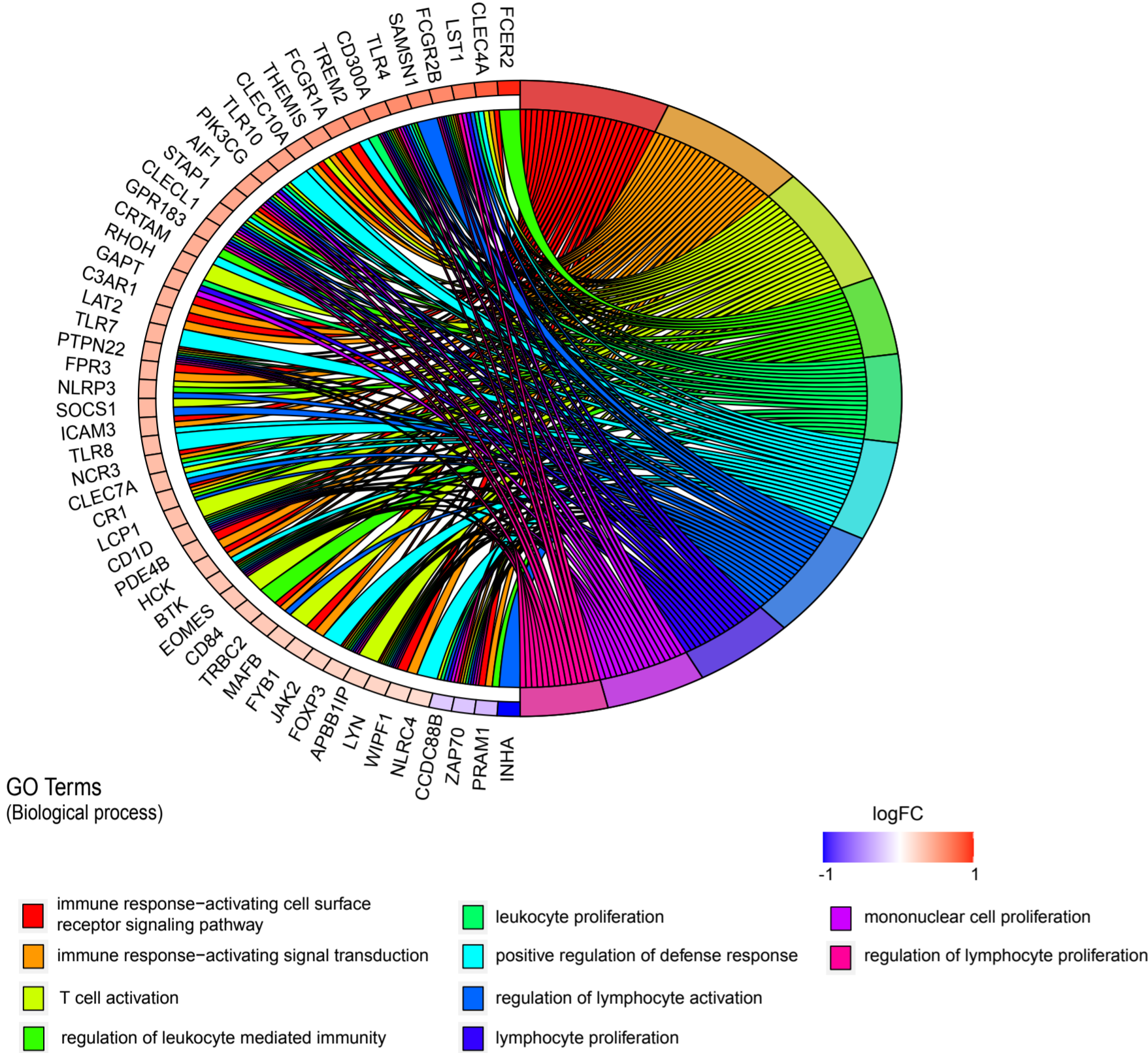

D

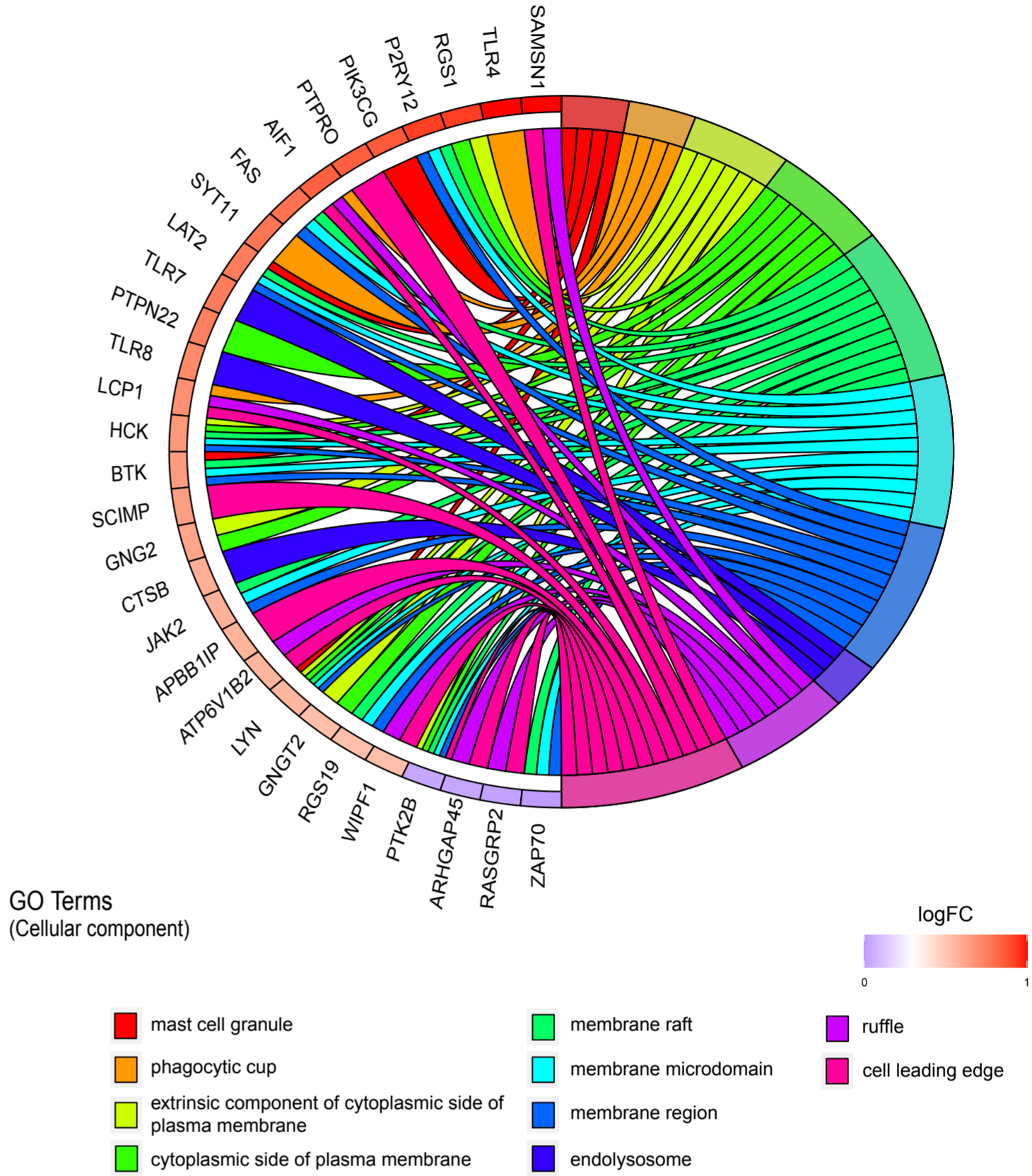

E

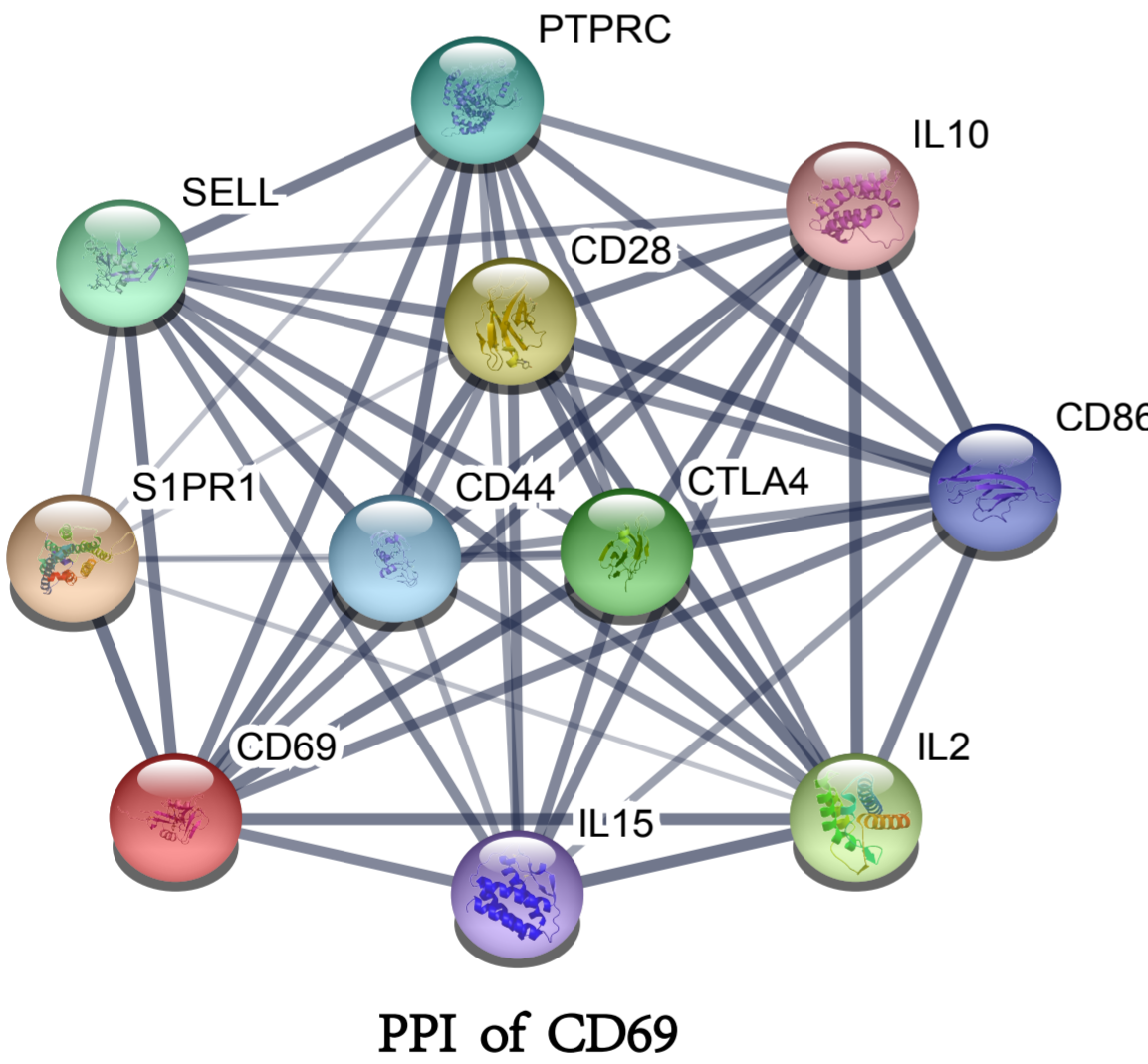

F

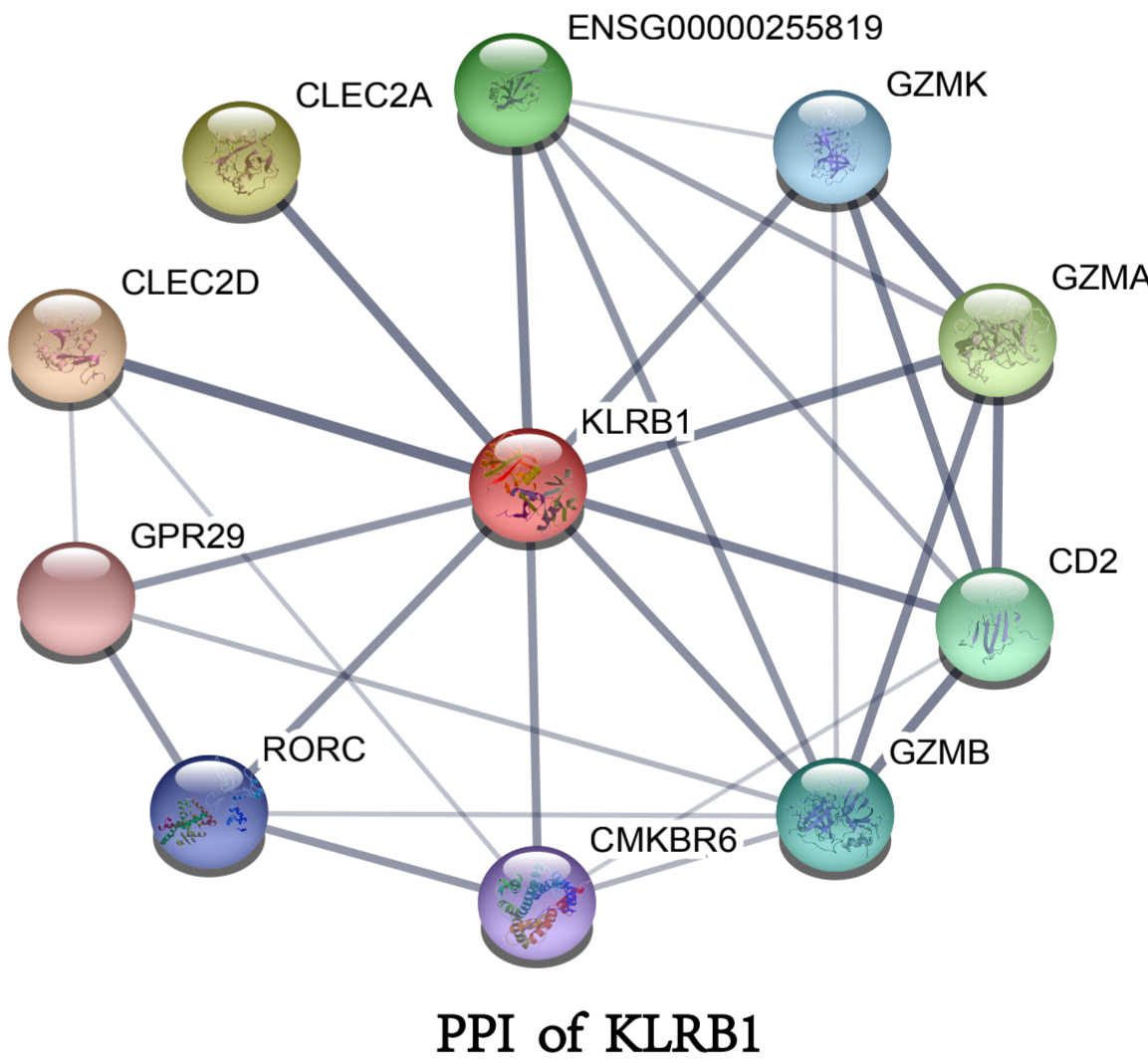

G

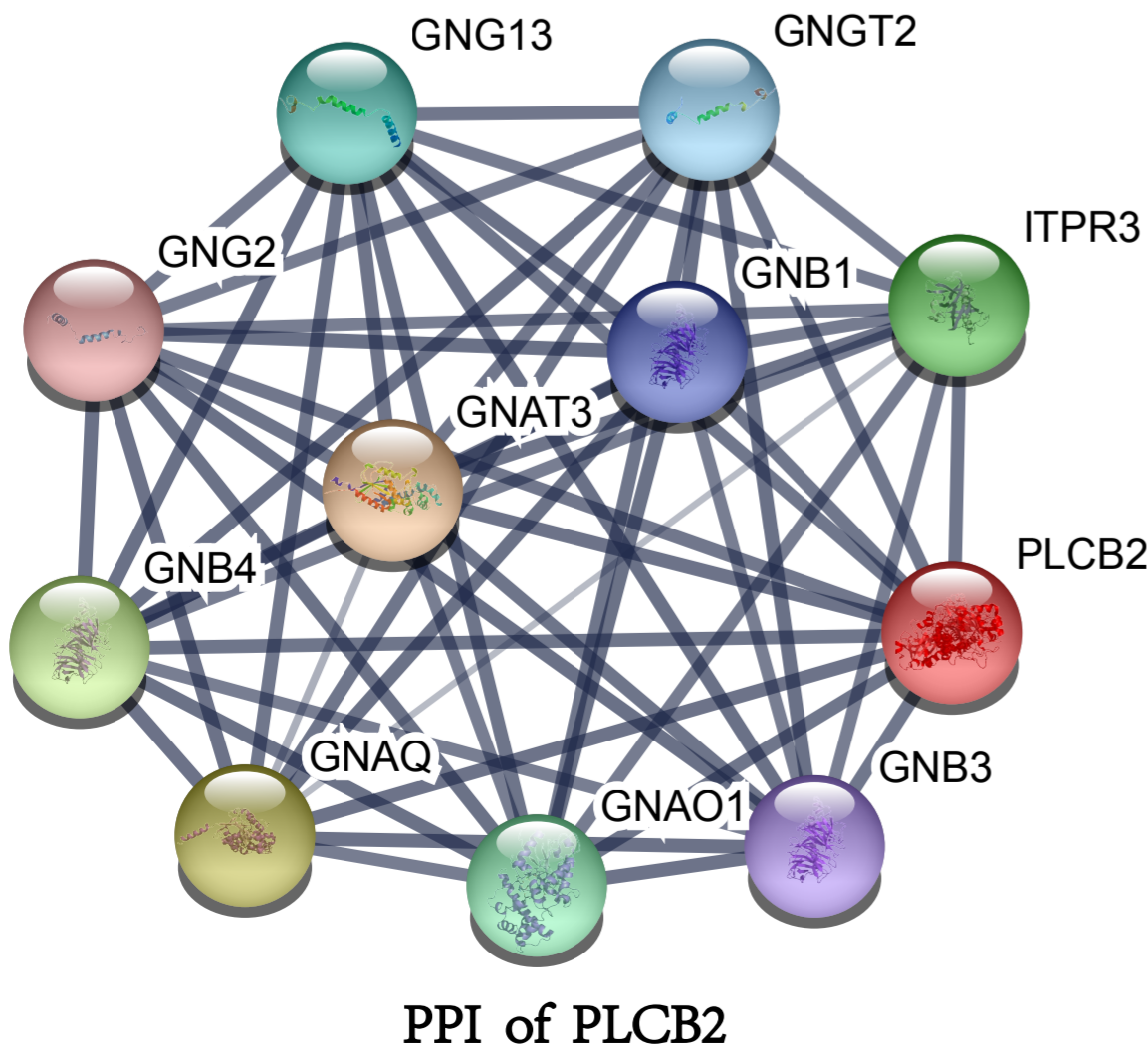

H

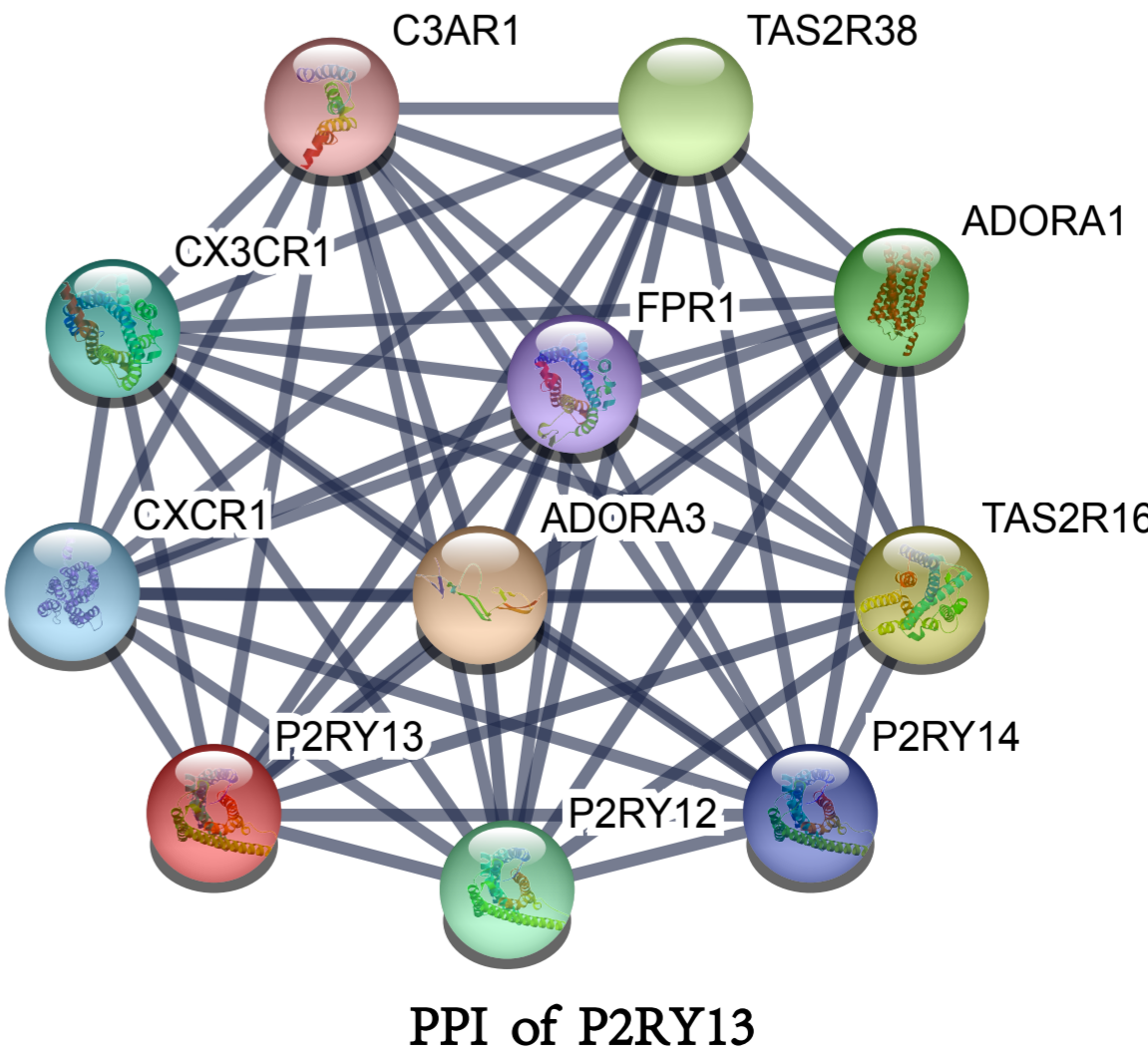

Supplement: Supplementary file 1 [file DataSheet_1.zip › Supplementary materials/Supplementary Figures/Figure-S3.pdf]

A

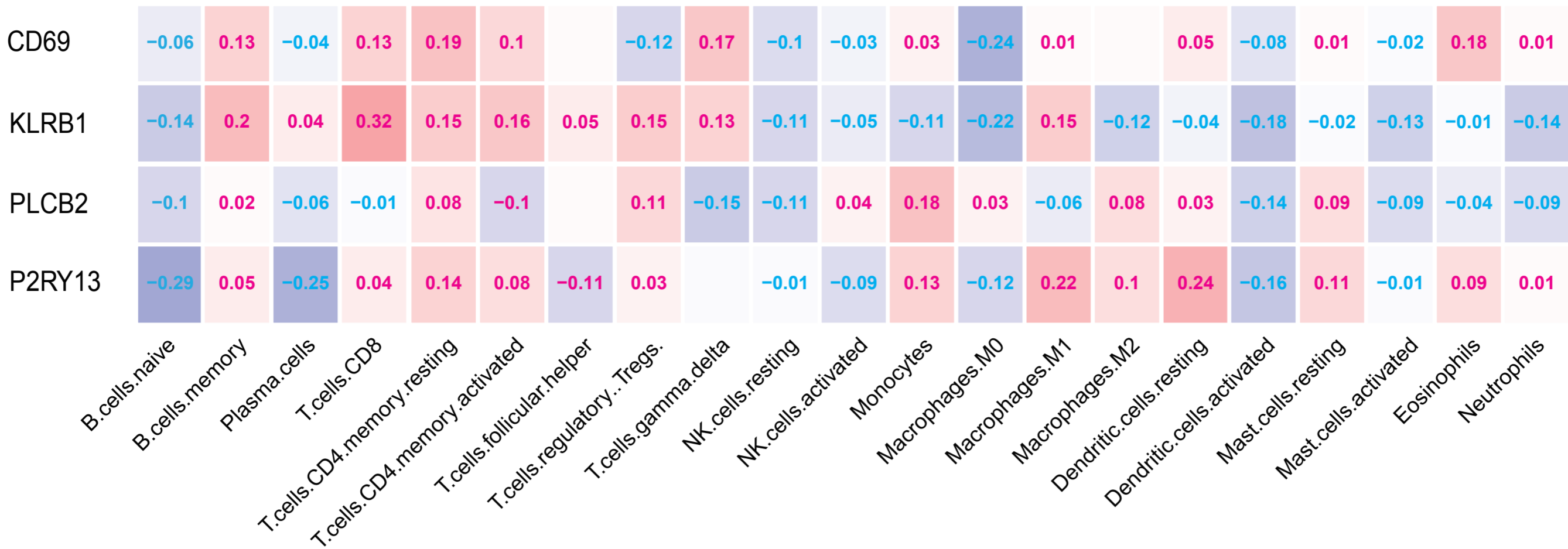

B

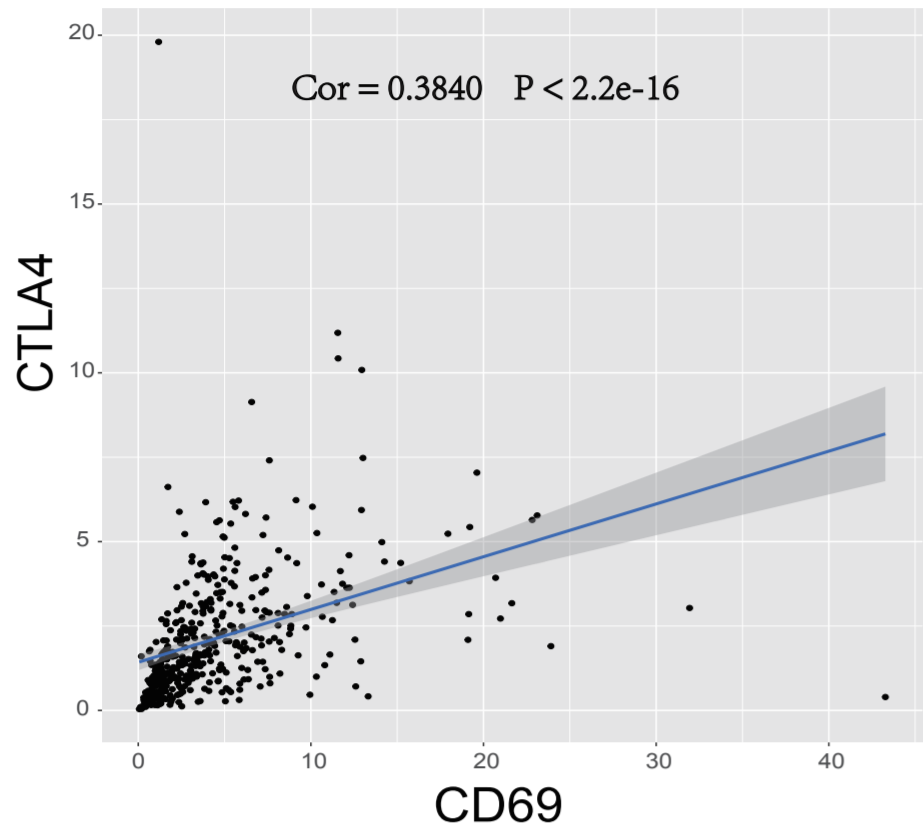

C

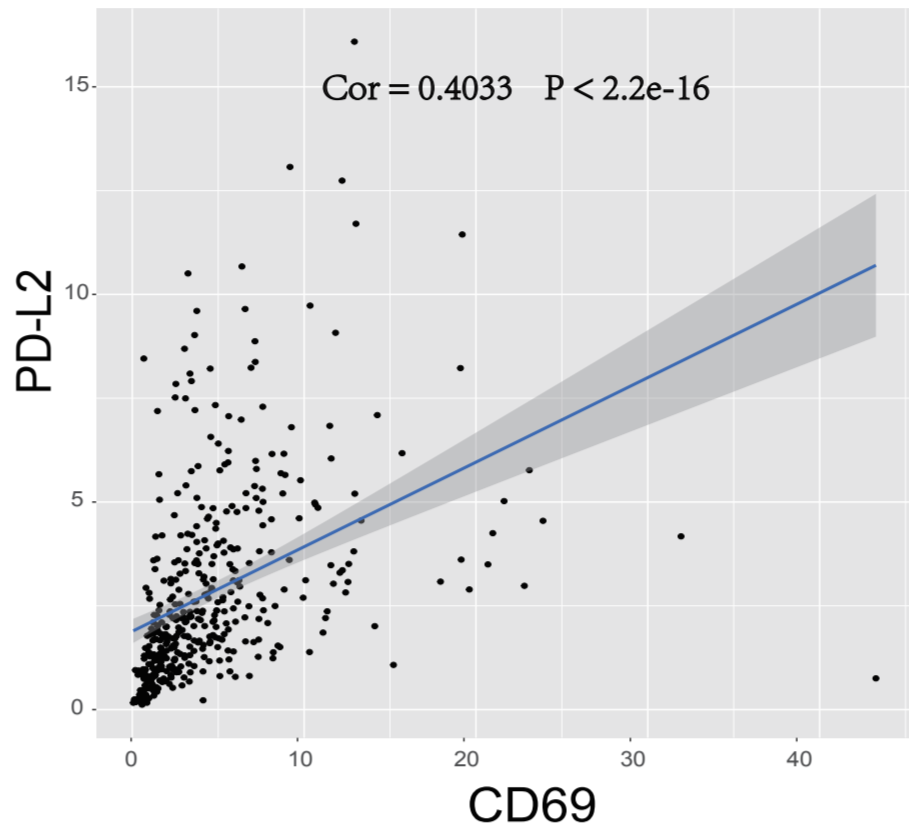

D

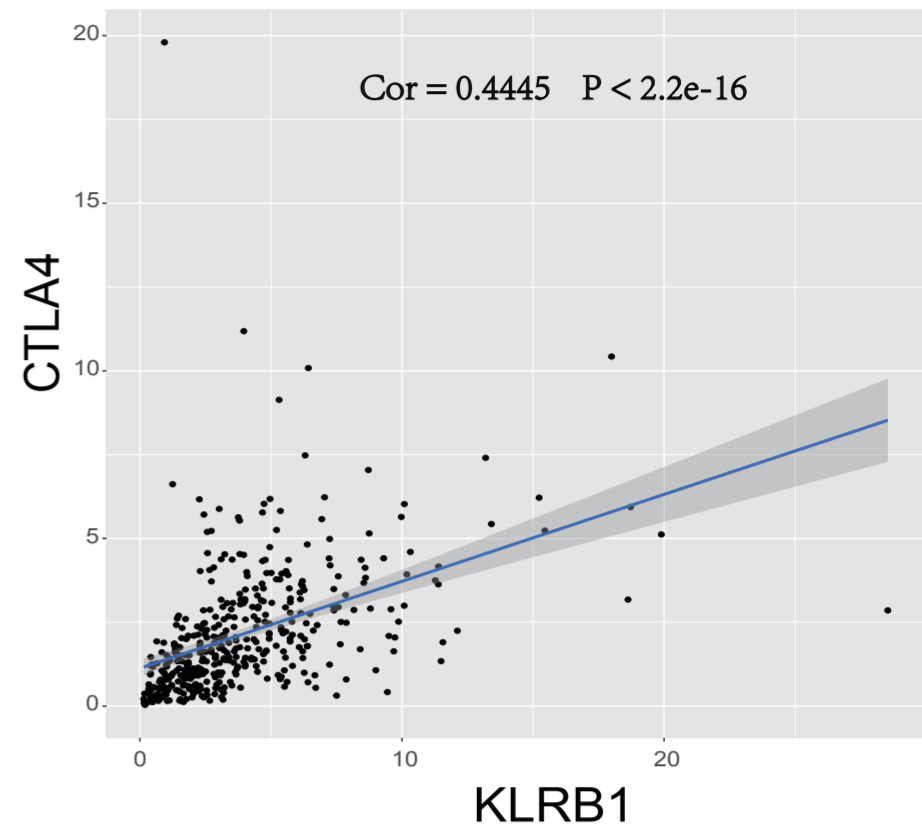

E

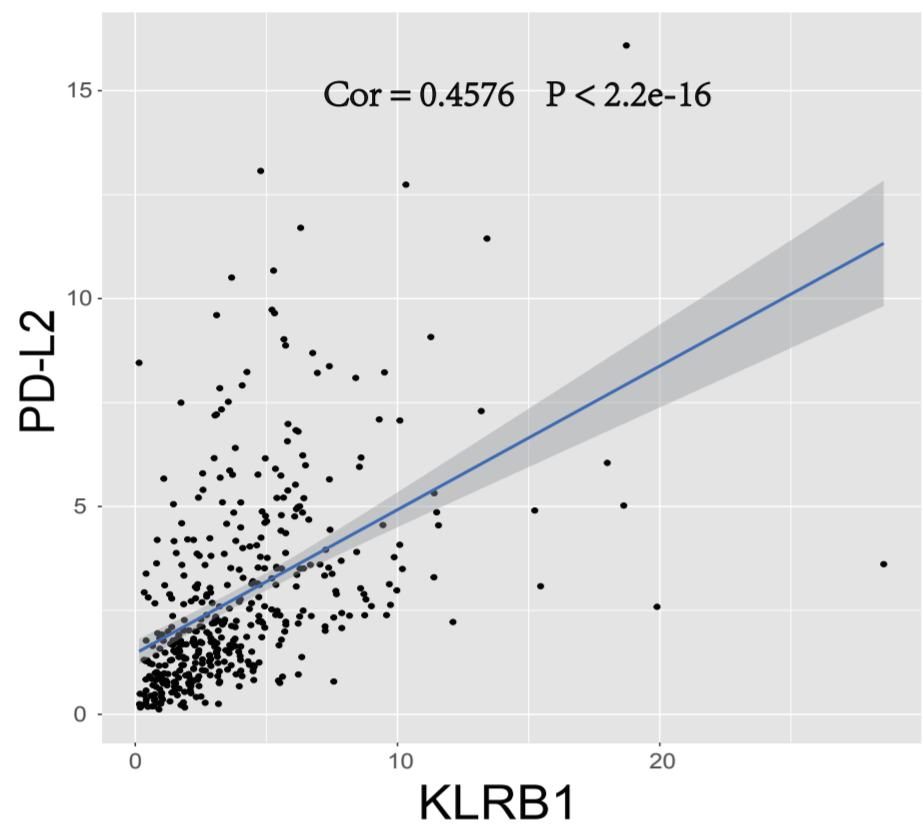

F

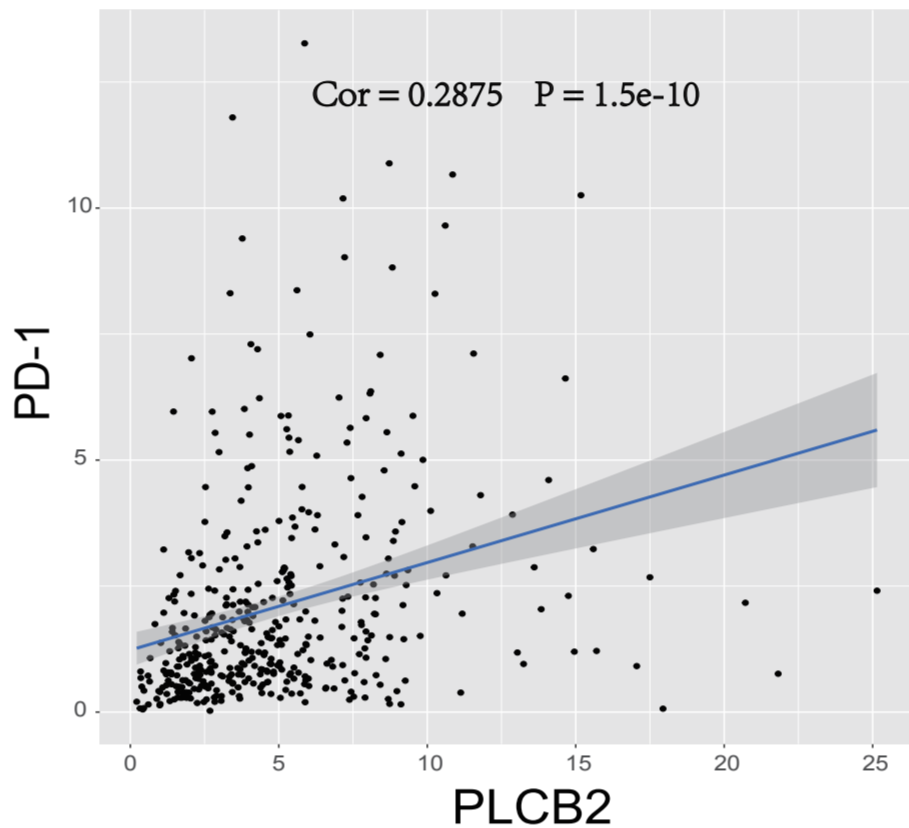

G

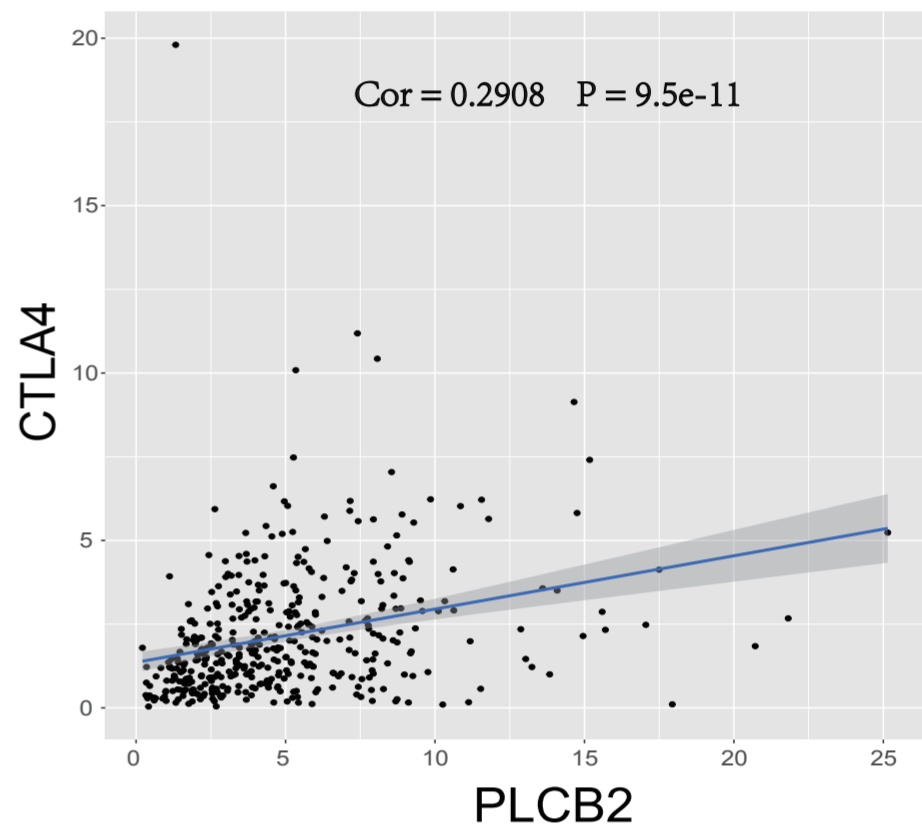

H

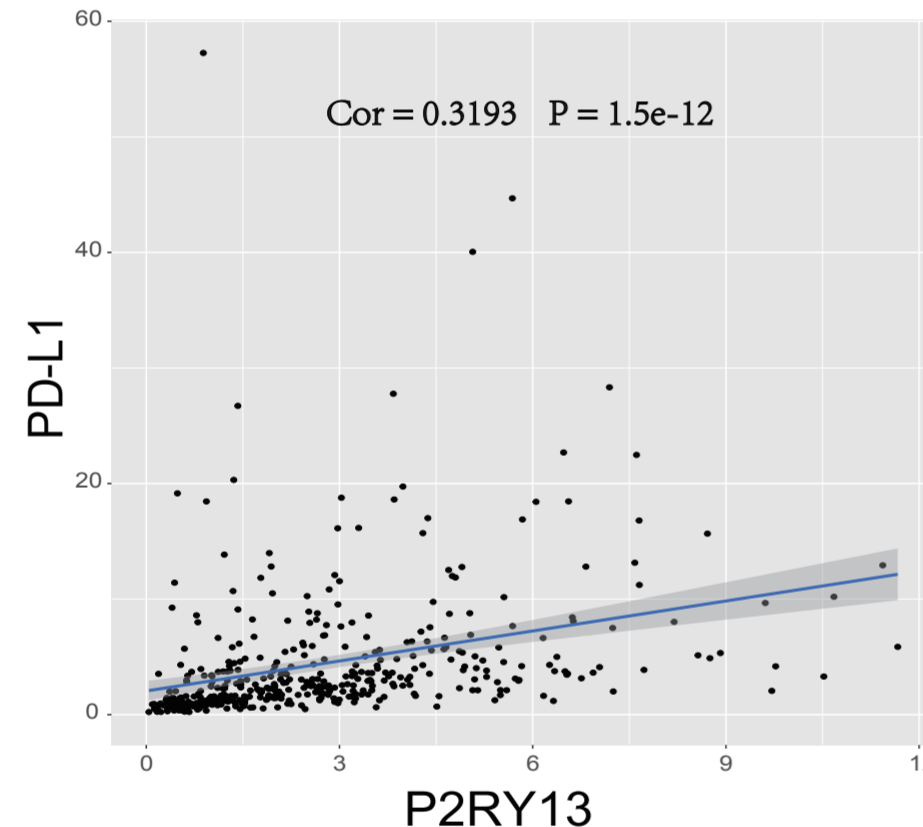

I

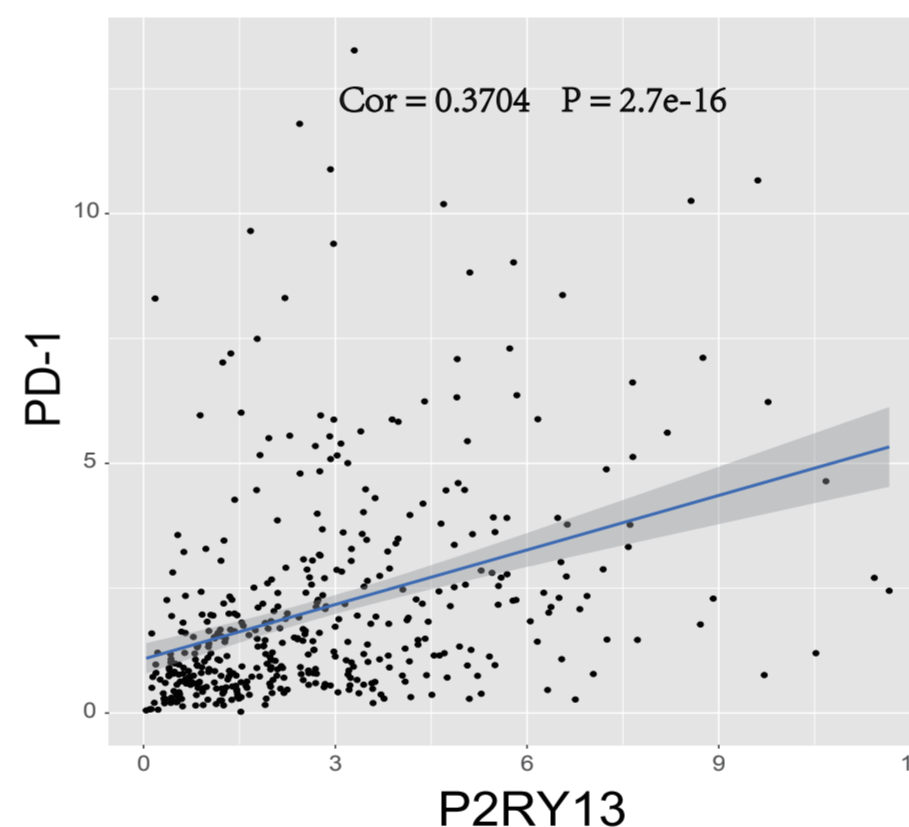

J

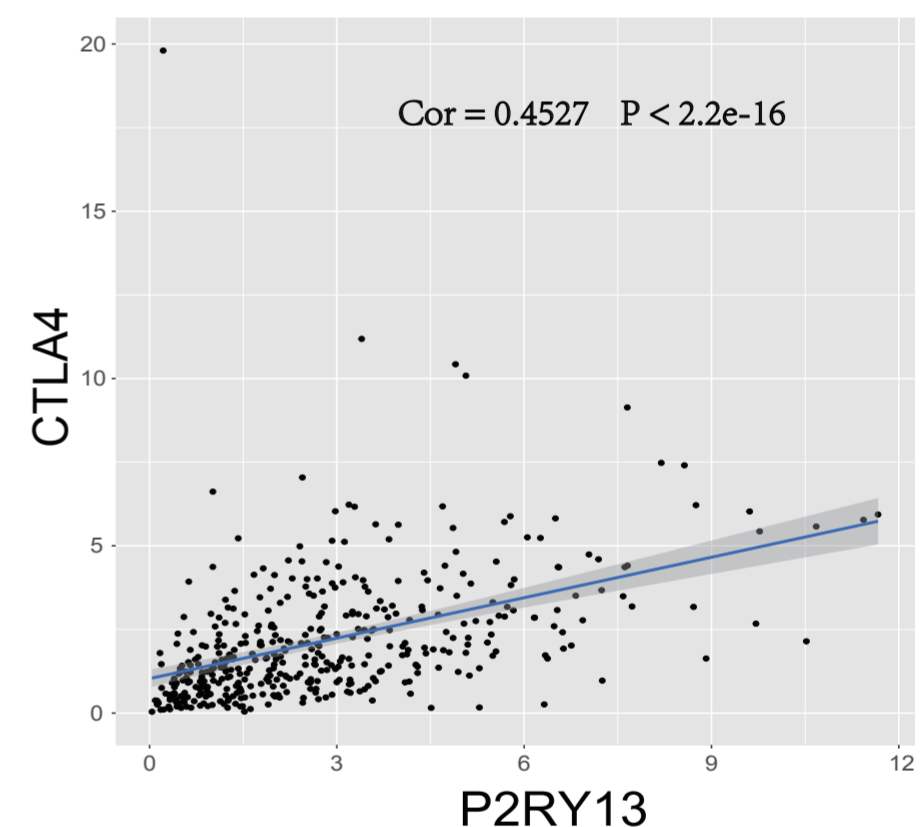

K

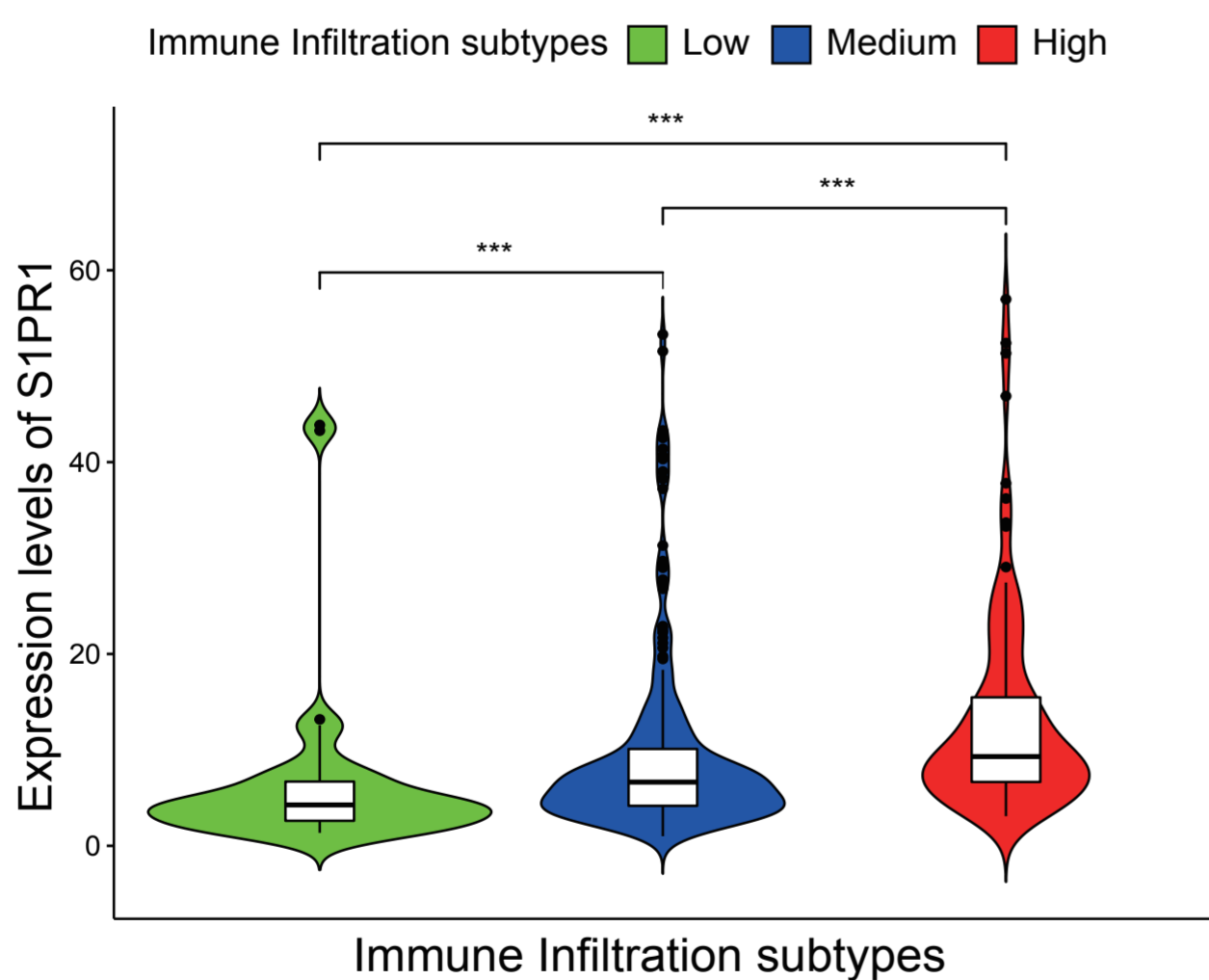

L

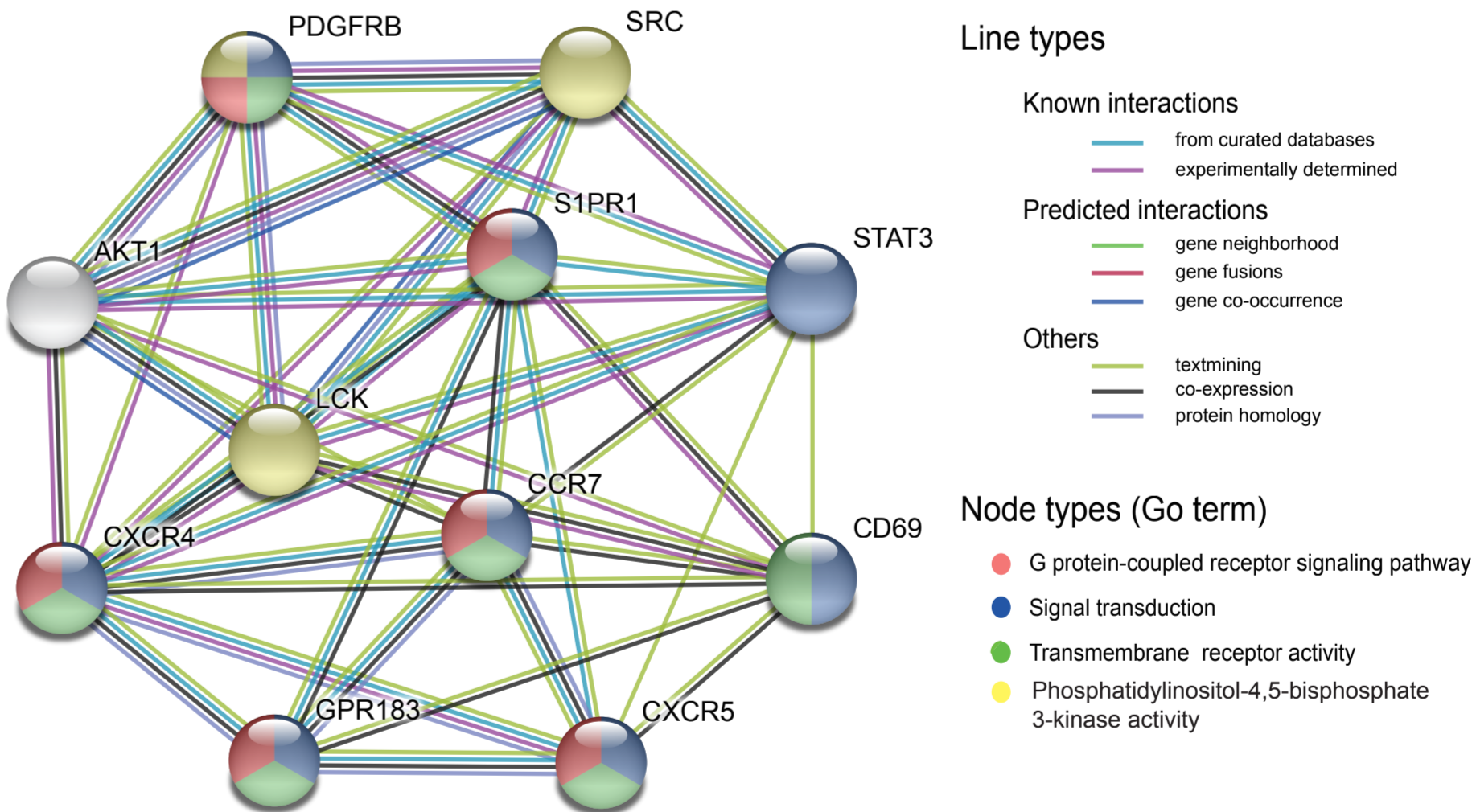

M

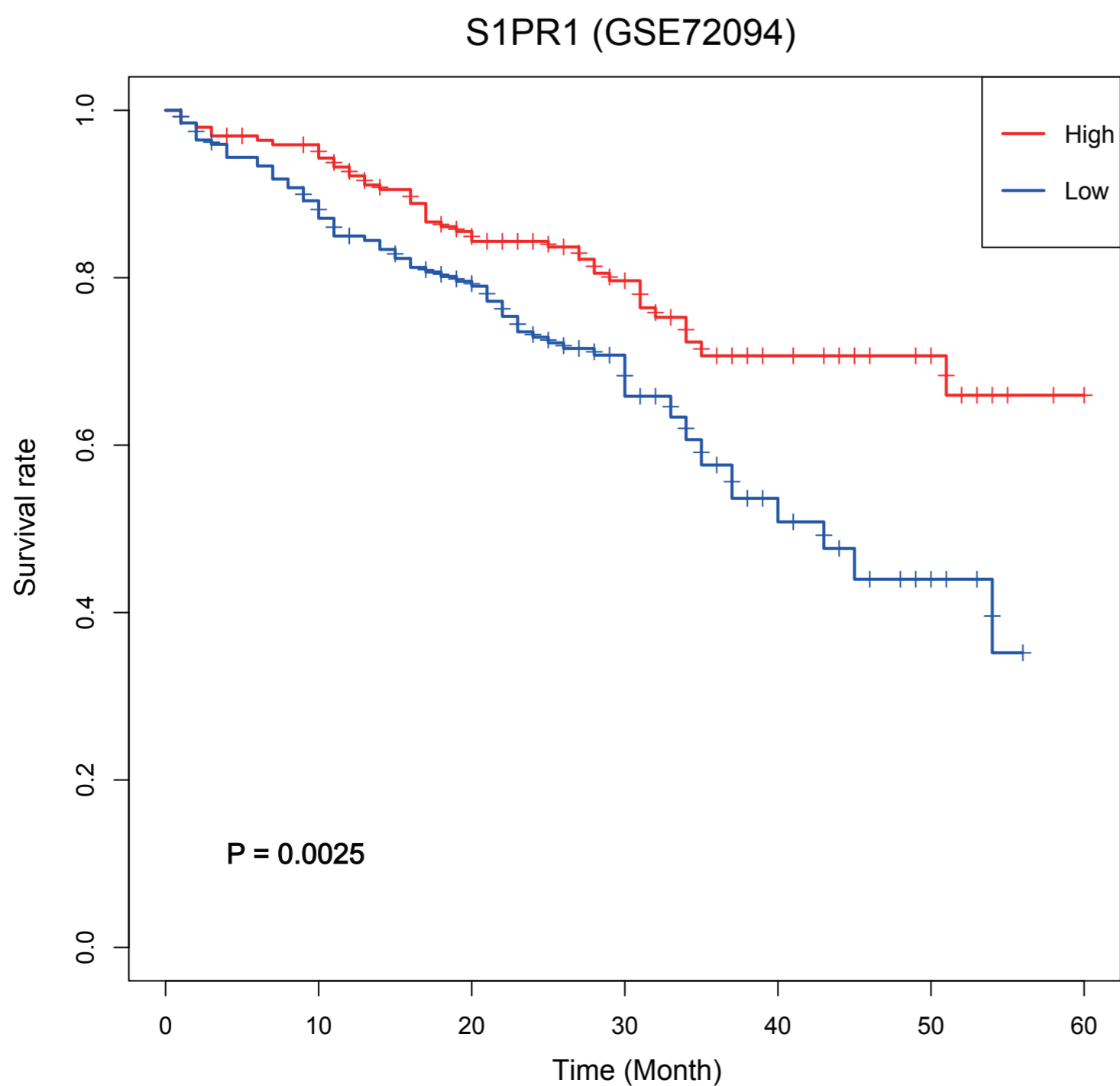

N

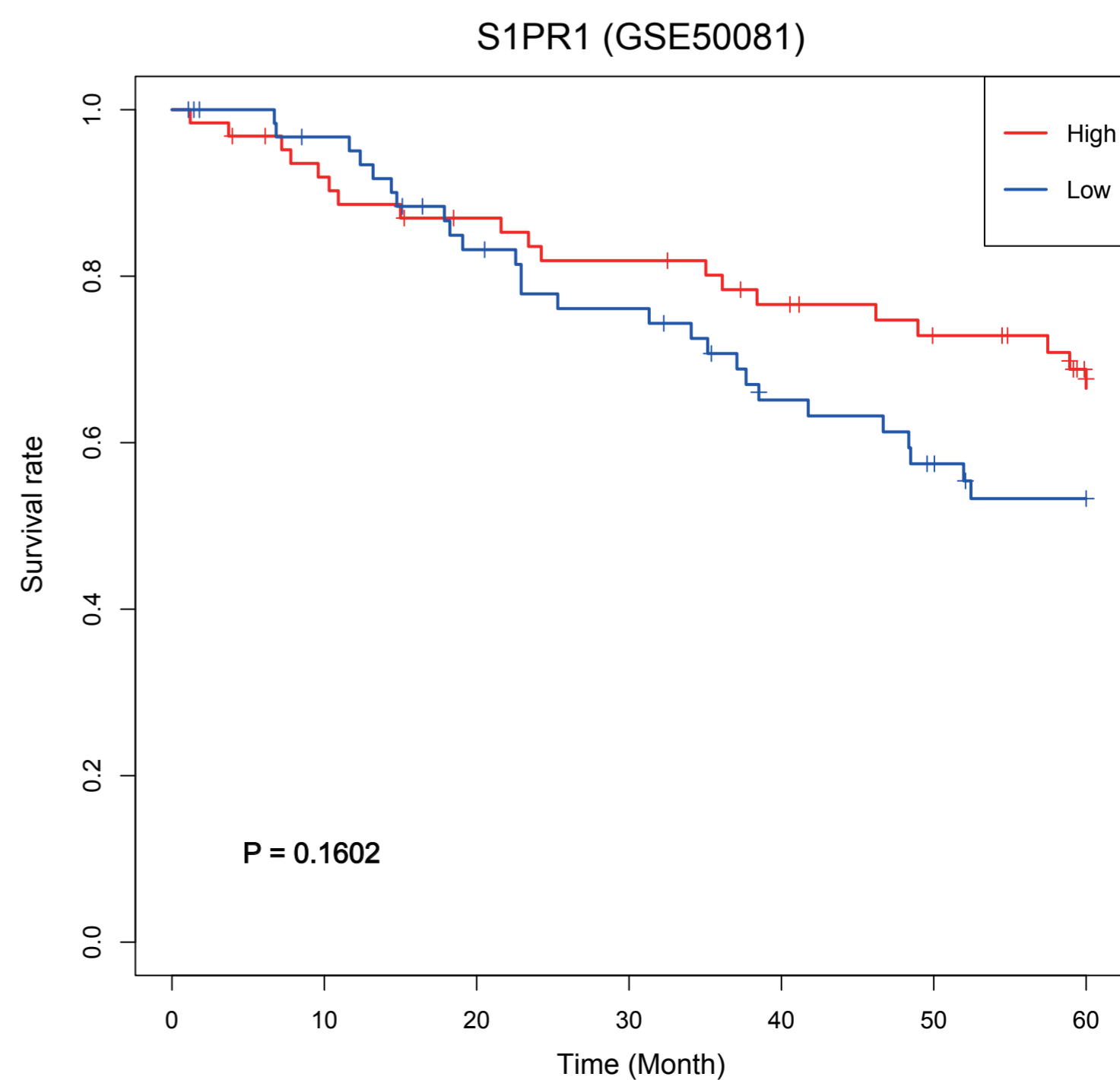

Supplement: Supplementary file 1 [file DataSheet_1.zip › Supplementary materials/Supplementary Figures/Figure-S4.pdf]

A

GSE 41271

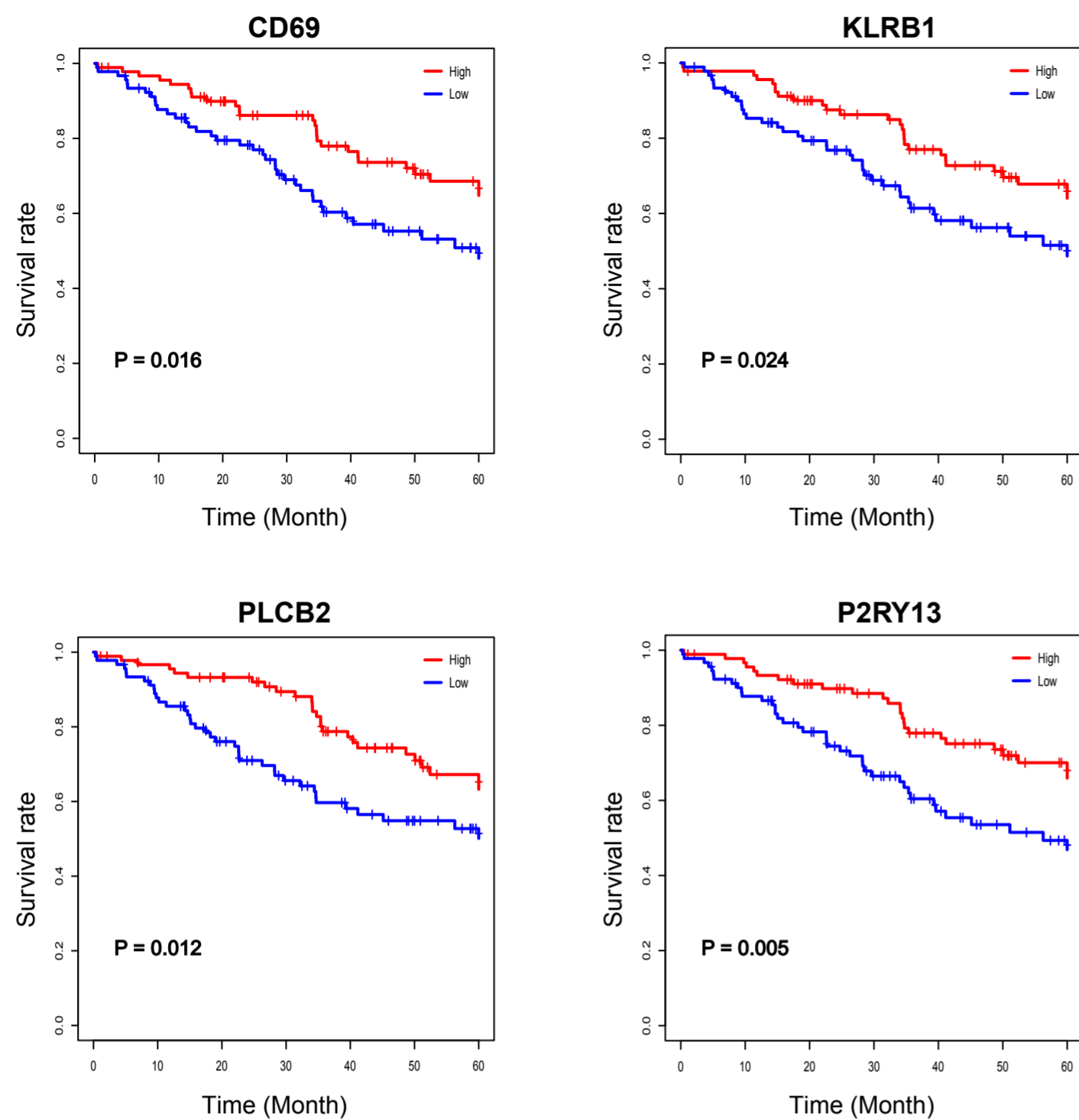

B

GSE 72094

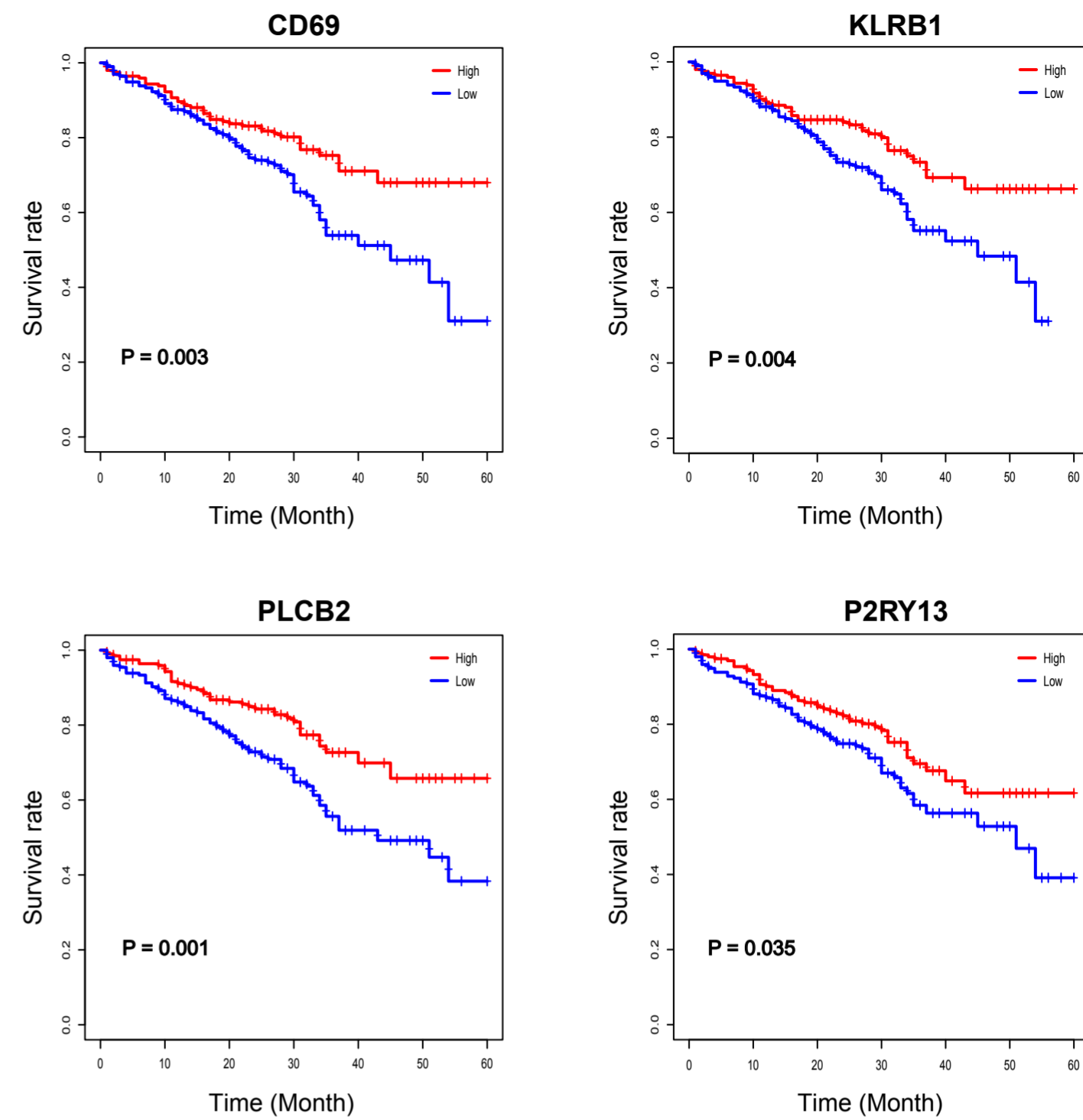

C

GSE 41271

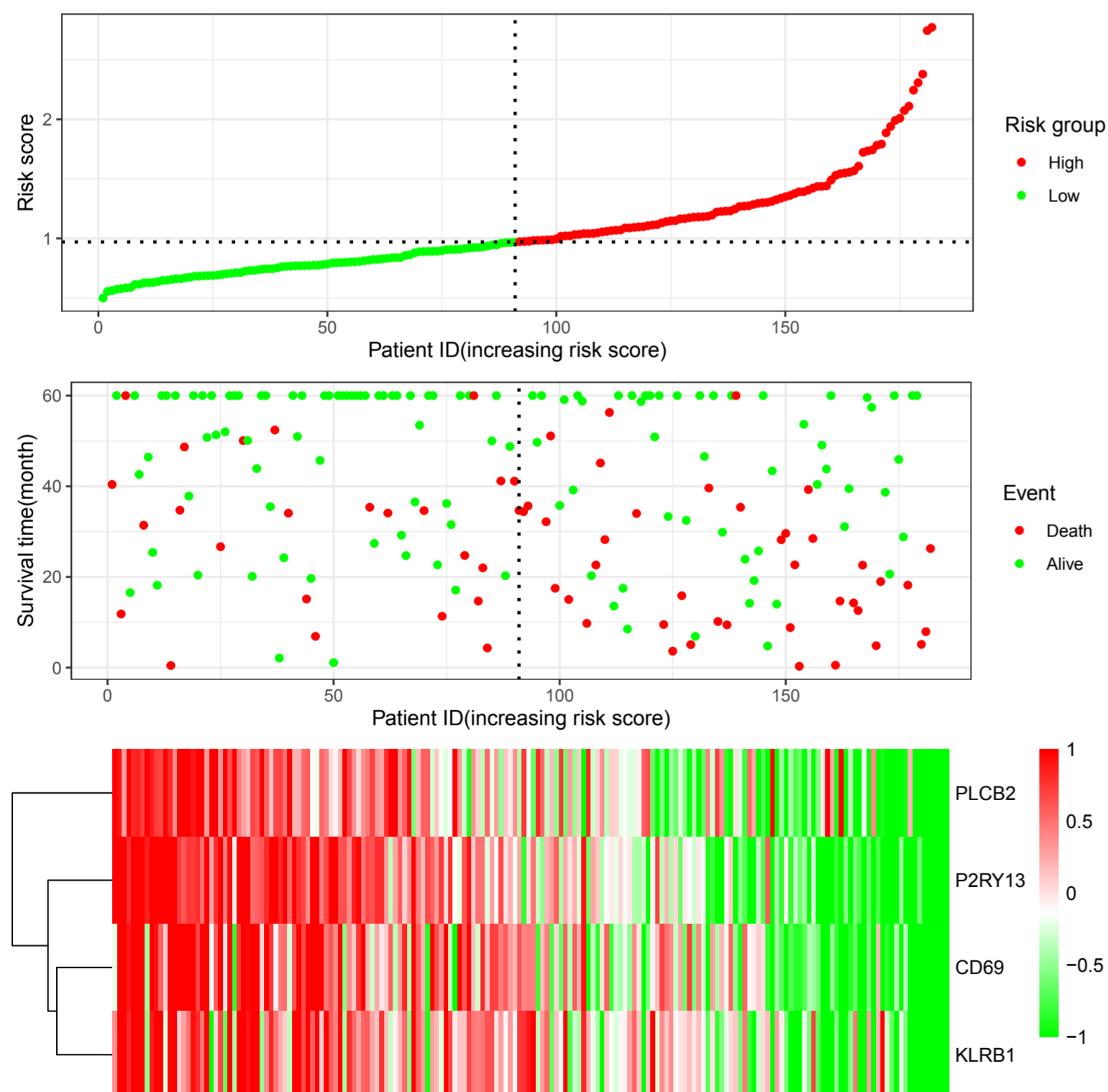

D

GSE 72094

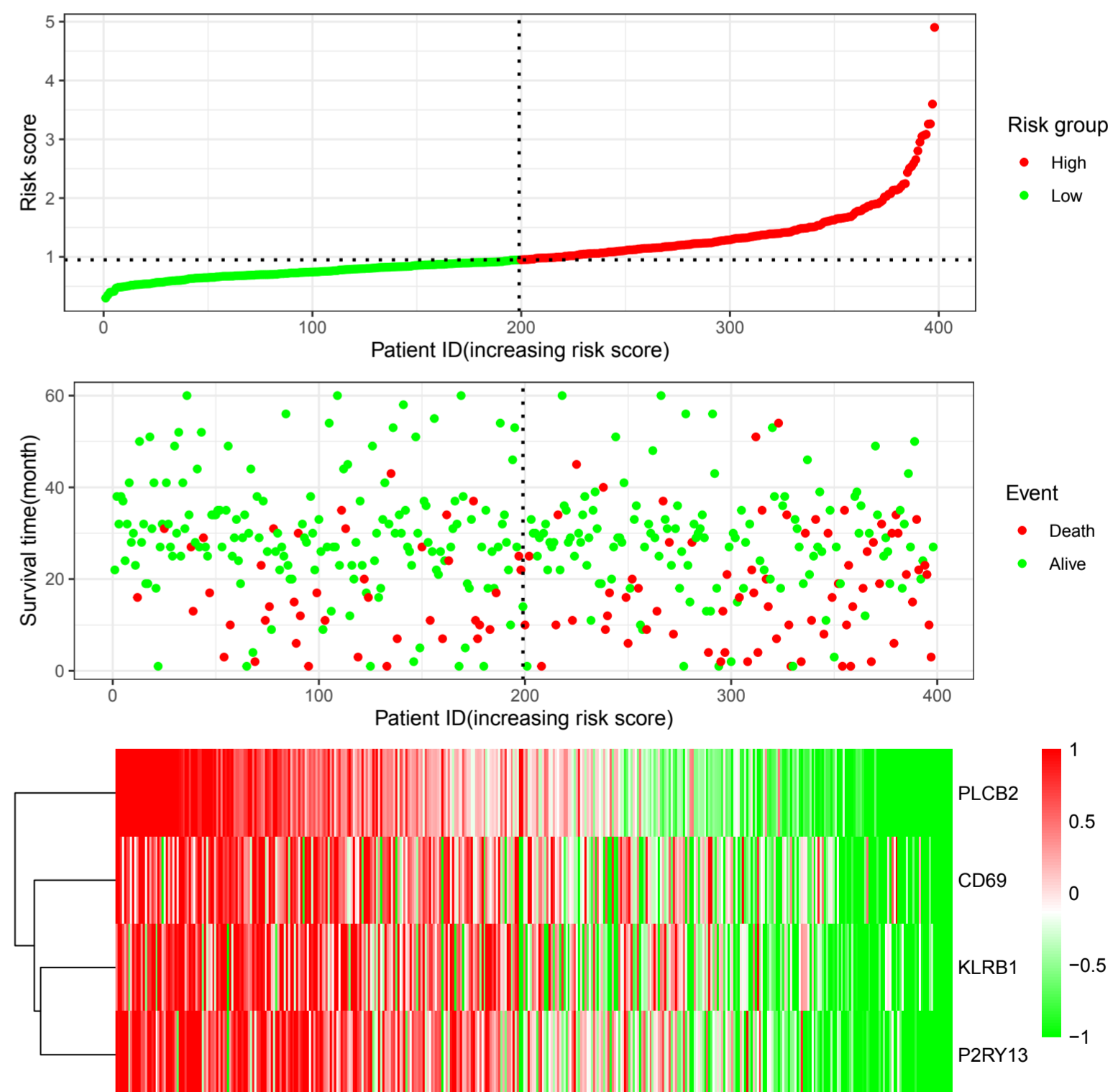

Supplement: Supplementary file 1 [file DataSheet_1.zip › Supplementary materials/Supplementary Figures/Figure-S5.pdf]
